# Phylogenetics of the Phlebotomine Sand Fly Group *Verrucarum* (Diptera: Psychodidae: *Lutzomyia*)

**DOI:** 10.4269/ajtmh.2011.11-0040

**Published:** 2011-06-01

**Authors:** Lee W. Cohnstaedt, Lorenza Beati, Abraham G. Caceres, Cristina Ferro, Leonard E. Munstermann

**Affiliations:** Arthropod-Borne Animal Disease Research Unit, Center for Grain and Animal Health Research, Agricultural Research Service, United States Department of Agriculture, Manhattan, Kansas; Georgia Southern University, Institute of Arthropodology and Parasitology, Statesboro, Georgia; Departmento Académico de Microbiologia Médica, Facultad de Medicina Humana, Universidad Nacional, Mayor de San Marcos, Lima, Peru; Laboratorio de Entomología, Instituto Nacional de Salud, Avenida Calle 26 No 51-60, Bogotá, Colombia; Instituto Nacional de Salud, Bogotá, Colombia; Yale School of Public Health, Yale University, New Haven, Connecticut

## Abstract

Within the sand fly genus *Lutzomyia*, the *Verrucarum* species group contains several of the principal vectors of American cutaneous leishmaniasis and human bartonellosis in the Andean region of South America. The group encompasses 40 species for which the taxonomic status, phylogenetic relationships, and role of each species in disease transmission remain unresolved. Mitochondrial *cytochrome c oxidase I* (*COI*) phylogenetic analysis of a 667-bp fragment supported the morphological classification of the *Verrucarum* group into series. Genetic sequences from seven species were grouped in well-supported monophyletic lineages. Four species, however, clustered in two paraphyletic lineages that indicate conspecificity—the *Lutzomyia longiflocosa–Lutzomyia sauroida* pair and the *Lutzomyia quasitownsendi*–*Lutzomyia torvida* pair. *COI* sequences were also evaluated as a taxonomic tool based on interspecific genetic variability within the *Verrucarum* group and the intraspecific variability of one of its members, *Lutzomyia verrucarum*, across its known distribution.

## Introduction

The New World sand fly genus *Lutzomyia* (Diptera: Psychodidae) encompasses approximately 391 species morphologically subdivided in subgenera and species groups.^1^,^2^ In northwestern South America, along the Andean mountain range, the *Verrucarum* species group includes eight of the principal sand fly vectors of American cutaneous leishmaniasis (ACL) and bartonellosis.^1^

The *Verrucarum* group consists of 40 species classified into three unranked series, *Serrana*, *Townsendi*, and *Verrucarum* (*sensu* Young and Duncan^1^), based on the phenotypic characteristics of the style and spines of the male genitalia. The *Verrucarum* series is characterized by two medial and two distal spines, the *Serrana* series is characterized by two distal spines and a slender medial spine, and the *Townsendi* series is characterized by three distal spines and an isolated basal spine.^3^ The females of the group are morphologically similar and difficult to identify reliably. A revision by Galati^4^ reorganized the group into seven series (*Monticola*, *Pacae*, *Pia*, *Evansi*, *Verrucarum*, *Serrana*, and *Townsendi*) based on a morphometric analysis of 88 morphological characters (two to six states per character).

Problems with morphological identifications in the *Verrucarum* group have led to difficulties in identifying leishmaniasis vectors,^5^,^6^ establishing their distributions,^7^ and clarifying their genetic relationships.^5^,^8^ The first attempt to move beyond classical taxonomy was the use of isozyme electrophoresis to distinguish members of the *Townsendi* series.^9^ Since then, species of the *Verrucarum* group have been subjected to taxonomic analysis by morphometrics,^10^ electron microscopy of eggs,^11^ karyotypic comparisons,^12^ and phylogenetic analysis of *cytochrome b* mitochondrial fragments.^5^ Galati's revision^4^ was given partial support by the analyses of 12S and 28S ribosomal DNA sequences,^13^ mitochondrial sequences,^5^ and nuclear isoenzymes.^6^ In these studies, phylogenetic relationships within the *Verrucarum* group remained, nevertheless, largely unresolved. In closely related taxonomic groups containing disease vector species, unambiguous species identification is necessary to ascertain the role of each species in disease transmission. Species identification within the *Verrucarum* group is unreliable when using female morphological structures.^5^ Consequently, the geographic limits of epidemiological risk cannot be delimited accurately,^7^ and planning meaningful control strategies becomes problematic. Furthermore, genetic data from each species will increase our understanding of the evolutionary relationships within the group,^8^ leading to a clarification of the evolutionary relationships between sand flies and the pathogens that they transmit.

*Cytochrome c oxidase I* mitochondrial (*COI*) sequences have been used to examine sibling species comparisons only within the *L. longipalpis* (Lutz and Neiva) complex.^14^ In the current study, *COI* sequences were used to reconstruct the phylogenetic relationships among the three *Verrucarum* group series (*sensu* Young and Duncan^1^). Furthermore, genetic variation of *L. verrucarum* (Townsend) was surveyed across its known range to assess its homogeneity as a single species. The use of these sequences as taxonomic identification tools is also investigated.

## Materials and Methods

**Figure 1.**
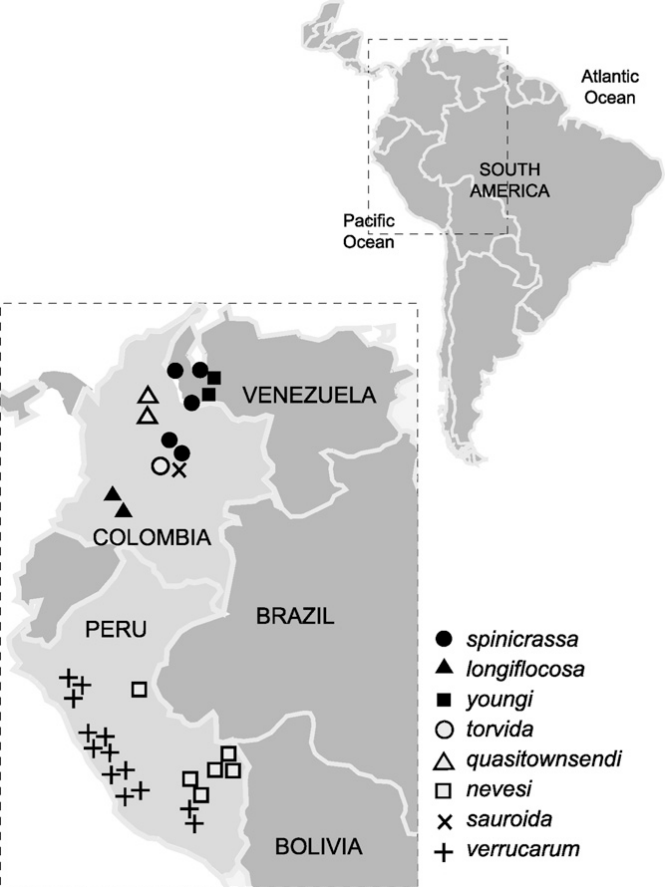
Distributions of the *Verrucarum* group sand flies used in the phylogenetic analysis. *L. serrana* (not shown) has a nearly continuous distribution throughout Peru, Ecuador, and Colombia. *L. robusta* is morphologically similar to *L. serrana* and reported in similar areas.

Between 2006 and 2008, 11 species of sand flies were collected in Peru and Colombia using standard white-light Centers for Disease Control and Prevention (CDC) traps as well as the light emitting diode (LED)-modified CDC traps described by Cohnstaedt and others^15^ (Figure 1). The *L. verrucarum* sensu stricto used in the intraspecific analysis was collected from six provinces—Amazonas, Cajamarca, Piura, Ancash, Lima, and Huancavelica (Tables 1 and 2). Geographic coordinates were collected using a Garmin Mark V GPS unit (datum from World Geodetic System 1984) and confirmed with Google Maps. *L. walkeri* (Newstead), assigned to the closely related subgenus *Migonei*, served as an outgroup. Only male sand flies were used in this study and were identified using the Young and Duncan taxonomic keys.^1^

DNA extractions and preparation for amplification have been previously described by Beati and Keirans.^16^ COI primers LCOI490 (5′-GGTCAACAAATCATAAAGATATTGG-3′) and HCO2198 (5′-TAAACTTCAGGGTGACCAAAAAATCA-3′) described by Folmer and others^17^ for metazoan invertebrates amplified approximately 700 sand fly bp, of which 667 were used. Polymerase chain reaction (PCR) conditions consisted of an initial denaturation at 95°C for 4 minutes followed by 20 cycles of denaturation (95°C) for 30 seconds, annealing (55–0.3°C/cycle) for 30 seconds, and elongation (70^o^C) for 35 seconds. The first 20 amplifications were followed by 25 additional cycles at 95°C for 30 seconds, 48°C for 1 minute, and 70°C for 35 seconds. The program was terminated with a 5-minute elongation step at 70°C. The PCR products were then purified by mixing with one part exonuclease I (NE Biol), antarctic phosphotase (NE Biol), one part PCR 10× buffer, and 17 parts biologically pure water, which was incubated sequentially at 37°C and 80°C for 15 minutes each. The purified sequences were bidirectionally sequenced at the DNA Analysis Facility, Science Hill (Yale University).

DNA sequences were aligned with a Clustal V algorithm using Lasergene software (DNAstar, Madison, WI). Base pair content and coding positions were computed using Mega 4.0.^18^ Kimura two-parameter (K2P) genetic divergences between DNA sequences were calculated using the computer program DNAsp.^19^ Model parameters for maximum likelihood phylogenetic tree construction were generated with Modeltest 3.7.^20^ The tree was generated by Bayesian analysis, Mr. Bayes 3.1,^21^ using the model parameters selected by Modeltest. One million iterations were performed on the data using four chains, and trees were recorded every 1,000th generation. A posteriori probability branch support was calculated by the 50% majority rule consensus of the recorded trees. The first 250 trees, recovered before the probability values converged and stabilized, were discarded before calculating posterior probabilities for each branch. To visualize the relationships between closely related haplotypes, a gene network was constructed using statistical parsimony.^22^

## Results

Eleven species from three series were recovered from the Peru–Colombia collections. The species from each series were as follows: (1) the *Verrucarum* series: *L. verrucarum*, *L. andina* (Osorno, Osorno-Mesa, and Morales), and *L. nevesi* (Damasceno and Arouck); (2) the *Serrana* series: *L. serrana* (Damasceno and Arouck) and *L. robusta* (Galati, Caceres, and LePont); and (3) the *Townsendi* series: *L. longiflocosa* (Osorno-Mesa, Morales, Osorno, and Munoz de Hoyos), *L. sauroida* (Osorno-Mesa, Morales, and Osorno), *L. torvida* (Young, Morales, and Ferro), *L. quasitownsendi* (Osorno, Osorno-Mesa, and Morales), *L. youngi* (Feliciangeli and Murillo), and *L. spinacrassa* (Morales, Osorno-Mesa, Osorno, and Munoz de Hoyos). The 56 specimens of *L. verrucarum* were collected from eight localities throughout the species range.

*COI* sequences were obtained from 72 specimens (Table 3). The alignment resulted in a 667 bp matrix, with 162 variable nucleotide positions (Table 4). Indels and nonsense/stop codons were not detected. The sequences were adenosine and thymine (AT)-rich, especially in the first and third codon positions (Table 5). All the base pair substitutions were synonymous in the *Verrucarum* series. Sequences of the two species of the *Serrana* series had amino acid changes in the alignment at positions 38 (valine to isoleucine) and position 482 (threonine to serine) relative to the base sequence of the *Verrucarum* series. In the *Townsendi* series, only *L. sauroida* had a serine to lysine amino acid change at position 369.

**Figure 2.**
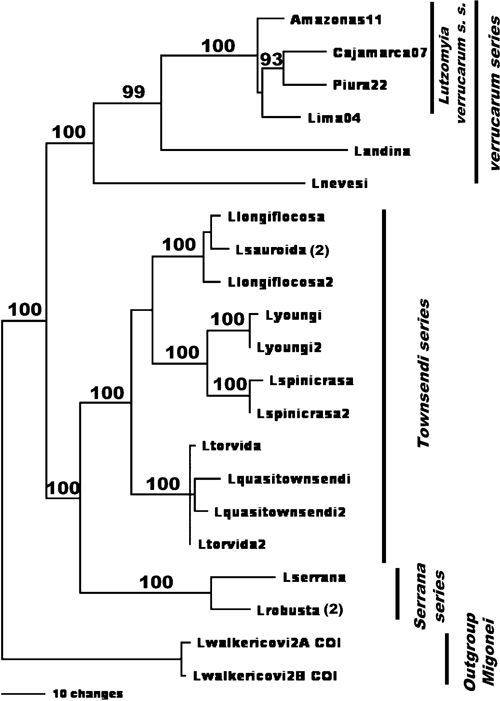
The phylogenetic tree was obtained by Bayesian analysis of the *COI* sequence. A posteriori probabilities are shown above branches. Species name and specimen code are included on the branch tips. The number of specimens with the same haplotype was listed in parenthesis.

Phylogenetic analysis of *Verrucarum* group species using Bayesian analysis indicated clear monophyletic clustering of the three series (Figure 2). Analysis parameters selected by Modeltest were GTR + I + G, shape = 1.74, and pinvar = 0.64. The deep rooted branch of the *Verrucarum* series indicated an early divergence from the sister clades corresponding to the *Serrana* and *Townsendi* series. Based on monophyly of each corresponding lineage, individual species assignments were made for seven species—*L. verrucarum*, *L. andina*, *L. nevesi*, *L. serrana*, *L. robusta*, *L. youngi*, and *L. spinicrassa*. Four species, *L. longiflocosa*, *L. sauroida*, *L. quasitownsendi*, and *L. torvida*, clustered as two paraphyletic clades.

**Figure 3.**
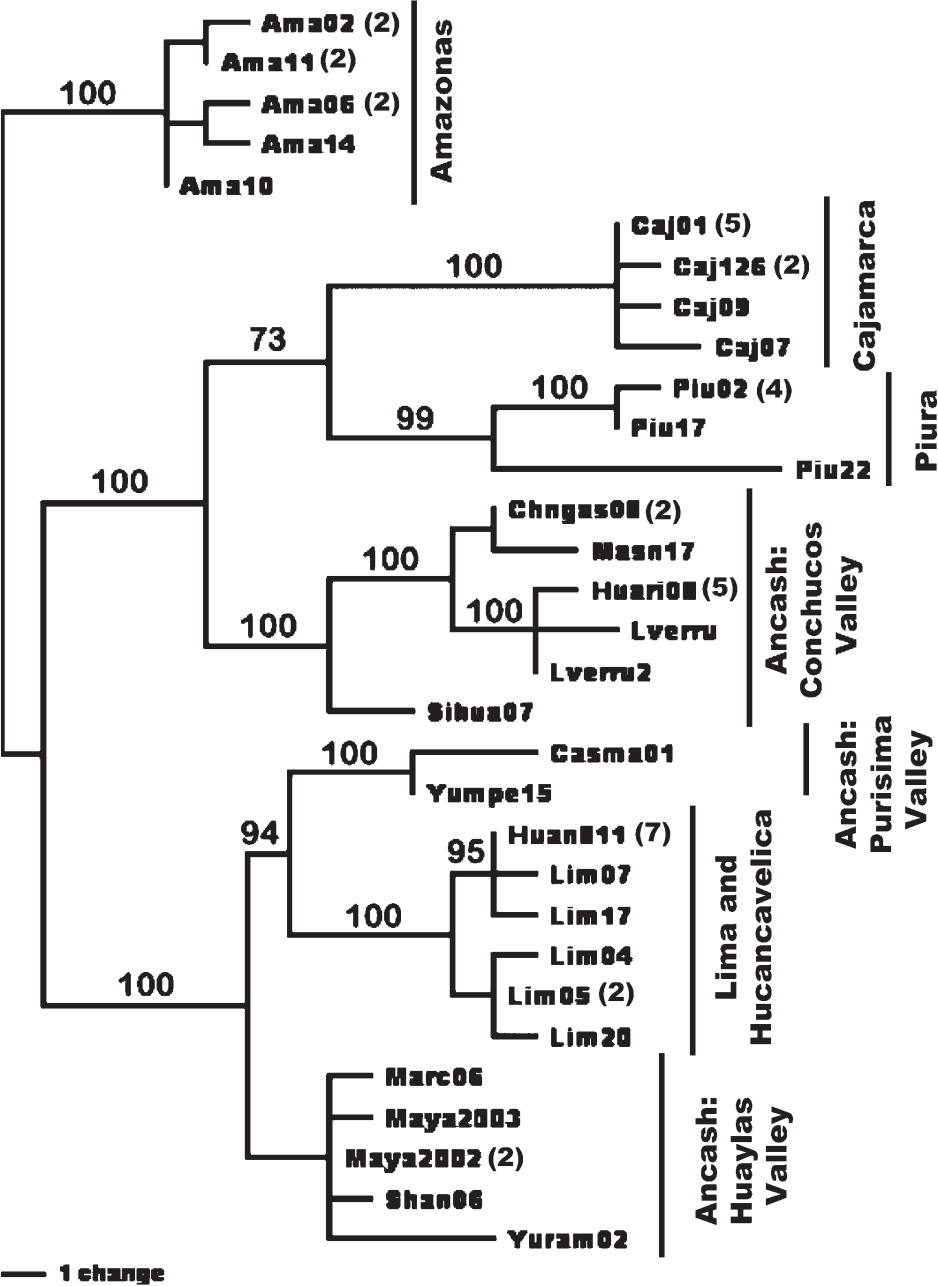
The phylogenetic tree was obtained by Bayesian analysis of *COI* sequence data, and a posteriori probabilities are shown above branches. Specimen name and code are listed on branch tips, and the number of specimens with the same haplotype was listed in parenthesis.

*L. verrucarum* s.s. phylogenetic analysis detected three main monophyletic clades: the Amazonas clade, the northeastern Andean clade, and the southwestern Andean clade (Figure 3). Bayesian analysis parameters selected by Modeltest were TVM + I + G, shape = 0.51, and pinvar = 0.60. The basal Amazonas clade encompassed eight specimens from the Amazonas province. The Amazonas clade diverged early from the northeastern and southwestern Andean clades.

The northeastern Andes clade consisted of three monophyletic clades. The Cajamarca and the Piura clades included the collections from the northern provinces Cajamarca (10 specimens) and Piura (6 specimens). The third clade, Ancash*-*Conchucos Valley, was formed by 18 specimens sampled within the interandean Conchucos Valley. The Sihua07 individual was collected from the town of Sihuas located at the northern collection site within the Conchucos Valley. Individuals from the other collection sites formed a single clade and were taken from the southern and southcentral parts of the valley. The southwestern clade consisted of three monophyletic clades. Collections from the Ancash*-*Huaylas Valley formed the most basal clade. The Ancash*-*Purisma Valley and Lima clade diverged from the Ancash*-*Huaylas Valley clade before separating. The Purisma Valley clade consisted of the northern-most (Casma01) and southern-most (Yumpe 15) collection points in the valley. The third clade consisted of samples from the southern-most provinces, Lima and Huancavelica. Sample Huan811, from the Huancavelica province, was located within the Lima clade.

**Figure 4.**
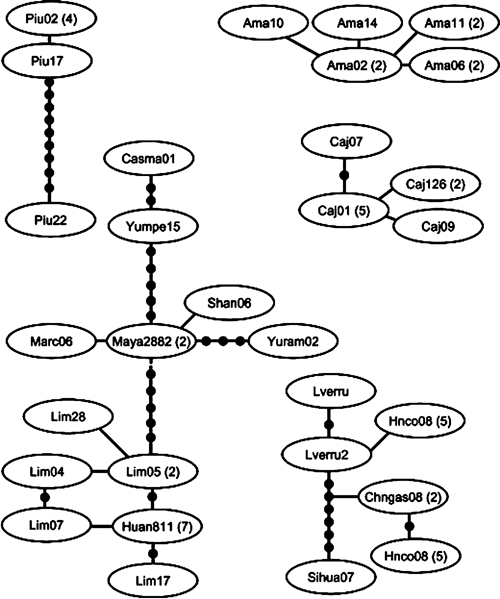
The geographic populations of *L. verrucarum* formed five unconnected genetic networks (based on statistical parsimony with a 95% connection limit) corresponding to the Amazonas, Cajamarca, Piura, Ancash (Conchucos Valley), and the western Andean populations. The western Andean network consisted of Ancash (Purisima and Huaylas Valleys), Lima, and Huancavelica populations. Species name and code number are listed in the ovals. Multiple identical haplotypes were indicated by parenthesis.

Genetic network analysis of the *L. verrucarum* geographic populations resulted in five unconnected networks using statistical parsimony with a 95% confidence limit (Figure 4). The eastern Andean populations each formed independent networks corresponding to Amazonas, Cajamarca, Piura, Ancash, and the Conchucos Valley. The eastern Andean population networks consisted of clusters of 1–2-bp variants, with the exception of Sihuas07 and Piura22. The western Andean population was a single connected network corresponding to Ancash, the Purisma and Huaylas Valleys, Lima, and Huancavelica. The northern/western Andean specimens from the Purisma Valley (Casma01 and Yumpe 15) were separated by 5 bp from the Huaylas Valley population. The Huaylas Valley population was 5 bp divergent from the Lima and Huancavelica populations.

Genetic divergence within the *Verrucarum* series was 14.9% between its three species, 5.2% between the six *Townsendi* species, and 4.2% between the two *Serrana* species. Intraspecific genetic variation among *L. verrucarum* s.s. geographic locations differed by 0.6–3.4% (Table 6). *L. verrucarum* s.s. from the western side of the Andes (Purisima, Huaylas, Lima, and Huancavelica) was more similar than populations from the eastern side (Conchucos, Amazonas, Cajamarca, and Piura). Genetic differentiation of the *L. verrucarum* s.s. by geographic locations ranged from 0.07 to 0.89.

## Discussion

The *COI* sequences can be an informative molecular tool for resolving phylogenetic relationships at the series and species level among recently evolved lineages within the *Verrucarum* group. However, comparisons of the genetic variability among and within series and species raise a number of questions about the taxonomic status of several species in this group. *COI* barcoding of sand fly species is appropriate for identifying potential disease vector specimens but must be used with caution when declaring new species designations based only on genetic divergence. Similar caution is necessary when using this fragment for phylogenetic reconstruction. Although apparently adequate for the purpose of the current study, the inclusion of one or more nuclear genes will increase the power of inferences drawn, because mitochondrial lineages have been shown to not always agree with nuclear gene lineages.

The *COI* primers amplified a single fragment in each sand fly. The absence of nonsense mutations in the alignment indicated an absence of pseudogenes. The AT-rich bias notable in the first and third codon position is common among invertebrates.^23^,^24^ Synonymous base pair mutations implied little phenotypic change and little or no selective pressure on the haplotypes. The three codons characterized by non-synonymous mutations, two in the *Serrana* series, maintained amino acid charge and polarity. The amino acid change in *L. sauroida* maintained polarity, but the amino acid charge became basic. Within the *Serrana* series, the amino acid changes were conserved in the two species and suggested a mutation early in the evolution of this series but after divergence from the *Townsendi* series. Within the *L. verrucarum* sequences, fixed base pair changes were found in geographically distinct populations, implying recent population level divergence.

The *COI* phylogeny indicated that the basal *Verrucarum* series lineage probably diverged before the *Townsendi* and *Serrana* series. This is the first molecular evidence of the ancestry and ranking within species of the *Verrucarum* group. The phylogenetic positions of three series (*Verrucarum*, *Serrana*, and *Townsendi: sensu* Young and Duncan^1^) in the *Verrucarum* group have been positioned unambiguously and support the taxonomic reorganization by Galati^4^ based on morphology. Young and Duncan^1^ and Galati^4^ placed 10 of 11 species treated herein within the same supported series. Only *L. nevesi* was moved to a newly formed series, the *Evansi* series in the Galati^4^ revision. The basal nature of *L. nevesi* and the high interspecies divergence between *L. nevesi* and the remaining species of the *L. verrucarum* series supported the inclusion of this species in a series distinct from *Verrucarum.* Additional species from Galati's^4^ *Evansi* series must be sampled to confirm this taxonomic separation, such as *L. evansi* (Nunez-Tovar), *L. marononensis* (Galati, Caceres, and Le Pont), and *L. ovallesi* (Ortiz).

*COI* sequences from 7 of 11 species formed monophyletic clades. The occurrence of two paraphyletic clades, the *L. sauroida–L. longiflocosa* and *L. torvida–L. quasitownsendi*, indicated that these four species require taxonomic reassessment. Based on enzyme electrophoresis results, Kreutzer and others^9^ suggested that *L. sauroida*, *L. longiflocosa*, and *L. quasitownsendi* may be conspecific. The current results support the conspecific relationship between *L. sauroida* and *L. longiflocosa*, and potentially, *L. quasitownsendi* based on the short branch lengths between clades. Similarly, *L. torvida* and *L. quasitownsendi*, species found in neighboring valleys, are morphologically similar and may form a cline of local variation. Nonetheless, *L. torvida* was distinguishable by diagnostic alleles with enzyme electrophoresis.^9^

Incomplete lineage sorting within the *Townsendi* series species can explain the close genetic similarities between species. Similarly, mitochondrial introgression has been reported between the species *L. townsendi* (Ortiz and Ortiz) and *L. youngi*^5^ and within the species complex *L. intermedia* (Lutz and Neiva).^25^ Additional specimens and species must be sequenced to better evaluate the taxonomic status of the clades. Increasing the number of species from the *Verrucarum*, *Serrana*, *Pia*, and *Evansi* series will also be necessary to clarify or support the phylogenetic relationships among the series as currently conceptualized as well as to evaluate the positions of the *Pia* and *Evansi* series within the phylogeny.

Phylogenetic analysis of *L. verrucarum* s.s. revealed a set of well-supported monophyletic clades that clustered by geographic collection area. Gene network analysis also supported the formation of separate clades as visualized by unconnected networks (Figure 4). The genetic distance among *L. verrucarum* populations indicated highly isolated populations, with little or no genetic exchange over extended periods of time. Analogous genetic structuring was observed in *L. shannoni* populations in Colombia^26^ and the *L. longipalpis* of Central and South America.^14^ The *L. verrucarum* populations in the northeastern Andes had a high genetic distance compared with those in the southwest. Sand flies are considered to have limited dispersal capabilities based on two relevant studies using New World sand flies. In mark–release–recapture experiments involving hundreds of flies, sand fly dispersal was estimated to be less than 200 m for *L. shannoni*^27^ and 500 m for *L. longipalpis*.^28^ Limited dispersal capability produces population structuring by distance, and the structuring is enhanced further in the presence of abiotic barriers. The heterogeneous terrain of the Andes, combined with the low dispersal capacity of the sand flies, has restricted their ability to colonize new areas and migrate between valley systems. In these conditions, allopatric speciation may progress rapidly to produce genetically distinct but morphologically similar individuals. The morphological homogeneity seen within species series (i.e., the *Townsendi* series^5^) or species complexes (*L. longipalpis*^29^) exemplifies this process. Furthermore, although separated by high mountains, the valleys of Ancash, Peru, are ecologically very similar. Their relatively homogeneous environmental conditions may have engendered little selective pressure on morphologically adaptive characters, thereby leading to cryptic speciation. However, careful transect sampling through potential dispersal routes within the Peruvian Andes and transpopulation mating experiments will provide the necessary additional information for species-level divergences implied by the current *COI* data.

Within the *Verrucarum* group, *Serrana* (4.2%) and *Townsendi* (5.2%) intraseries genetic divergence was similar to the intraspecific genetic divergence of *Anopheles annulipes* sensu lato (Walker; mean = 4%, range = 0.3-6.8%).^30^ The 17 morphologically similar sibling species of the *A. annulipes* species complex is a case of extreme diversification, with nearly complete isolation on the island continent of Australia. In contrast, the *Verrucarum* series divergence was threefold higher (14.9%). These comparisons may be somewhat misleading, because *L. nevesi* included as a *Verrucarum* series member has also been classified as part of the *Evansi* series.^4^ Furthermore, *L. verrucarum* sample numbers were greater than the other sand fly specimens, and the samples may have included an undescribed species complex. Additional specimens from several geographic sites for species in the *Serrana* and *Townsendi* series may increase intraseries divergence values and possibly, monophyly of species.

Genetic divergence (0.1–3.6%) within geographic populations of *L. verrucarum* was similar to that of species within the *A. annulipes* s.l. complex (mean = 0.4%, range = 0.0–2.0%).^30^ Under closer scrutiny, morphological traits may be discovered that will distinguish the isolated *L. verrucarum* valley populations as different taxa. This has precedence in other phlebotomine sand fly species, such as in clusters formerly regarded as single species (i.e., *L. longipalpis*)^31^ and the eight species of the *Townsendi* series.^1^,^5^ Taxonomy of the *Townsendi* series in Colombia has been emphasized, because four of these species (*L. quasitownsendi*, *L. spinacrassa*, *L. longiflocosa*, and *L. youngi*) are major vectors of leishmaniasis and must be distinguished from the non-vector species.^8^

The high genetic variability within *L. verrucarum* populations emphasized the importance of testing several sand fly specimens from diverse geographic locations. Although the geographic range of most species used herein was small (Figure 1), genetic differentiation reported within the limited habitat of *L. verrucarum* was high. The phylogenetic relationships among the species of the *Verrucarum* group (other than *L. verrucarum* s.s.) were based on few individuals from single geographic areas. Adding species, specimens, and geographic locations will be necessary to capture the genetic variability within the sand fly species if *COI* comes to be used in a barcoding capacity. *COI* barcoding has served as a rapid and reliable means of sampling morphologically similar species for genetic variability. Additionally, Besansky and others^32^ justified barcoding for species classification because of the importance of identification for medically important insects, such as sand flies. If all *COI* sequences have evolved at a uniform rate within the *Verrucarum* group, the representation of the *Townsendi* series main lineages as different species implies that the populations of *L. verrucarum* form a species complex because of a comparable genetic divergence. Conversely, if *L. verrucarum* is considered a single species, then the species currently included in the *Townsendi* series have a similar conspecific status. In both cases, the high genetic divergence between geographic populations of *L. verrucarum* s.s. requires resolution and may preclude the use of *COI* sequences as a barcoding tool for the description of new species and potentially, for the identification of sand flies.

In summary, phylogenetic analyses of *COI* sequences provided an initial well-supported reconstruction of the genetic relationships between sand fly lineages of recent origin. Nevertheless, the evolutionary history of the *Verrucarum* group species will be better resolved when representatives of all species and series are included in the same phylogenetic tree as well as specimens from diverse sites within their geographic distribution.

## Figures and Tables

**Table 1:** *Verrucarum* group sand fly species used in the phylogenetic analysis

Series	Species	No. samples	Country	Province	Accession numbers
*Verrucarum*	*L. andina*	1	Colombia	Cundinamarca	FJ437283
*Verrucarum*	*L. nevesi*	1	Colombia	Meta	FJ437270
*Serrana*	*L. serrana*	2	Colombia	Boyacá	FJ437274
*Serrana*	*L. robusta*	2	Peru	Cajamarca	FJ437280
*Townsendi*	*L. longiflocosa*	2	Colombia	Huila	FJ437271; FJ437273
*Townsendi*	*L. quasitownsendi*	2	Colombia	Boyacá	FJ437276; FJ437279
*Townsendi*	*L. sauroida*	2	Colombia	Boyacá	FJ437284
*Townsendi*	*L. spinicrassa*	2	Colombia	Boyacá	FJ437278; FJ437282
*Townsendi*	*L. torvida*	2	Colombia	Cundinamarca	FJ437275; FJ437281
*Townsendi*	*L. youngi*	2	Colombia	Mateguadua	FJ437272; FJ437277
*Lutzomyia (Migonei)*	*L. walkeri*	1	Colombia	Huila	FJ437285; FJ437286

**Table 2:** *Lutzomyia verrucarum* s.s. specimens from Peru

Province	Haplotype name	Accession nos.	Province	Haplotype name	Accession nos.
Huaylas	Huari08	FJ437253	Amazonas	Ama02	FJ437240
Huaylas	Marc06	FJ437259	Amazonas	Ama06	FJ437241
Huaylas	Maya2883	FJ437262	Amazonas	Ama10	FJ437242
Huaylas	Shan06	FJ437266	Amazonas	Ama11	FJ437243
Huaylas	Yuram01	FJ437268	Amazonas	Ama14	FJ437244
Huaylas	Yuram02	FJ437269	Cajamarca	Caj01	FJ437245
Purisima	Casma01	FJ437249	Cajamarca	Caj07	FJ437246
Purisima	Yumpe15	FJ437267	Cajamarca	Caj09	FJ437247
Conchucos	Chngas11	FJ437250	Cajamarca	Caja05	FJ437248
Conchucos	Hnco02	FJ437251	Piura	Piu02	FJ437263
Conchucos	Masin15	FJ437260	Piura	Piu17	FJ437264
Conchucos	Masn17	FJ437261	Piura	Piu22	FJ437265
Lima	Lim04	FJ437254	Lima	Lim17	FJ437257
Lima	Lim05	FJ437255	Lima	Lim28	FJ437258
Lima	Lim07	FJ437256	Huancavelica	Huan811	FJ437252

**Table 3:** Geographic locations of *L. verrucarum* specimens from Peru

Province	Region	Township	Latitude	Longitude	Elevation (m)
Ancash	Purisima	Casma	9° 29′ 35″ S	77° 59′ 37″ W	1,580
Ancash	Purisima	Yumpe	10° 15′ 29″ S	77° 29′ 13″ W	2,083
Ancash	Huaylas	Yuracmarca	8° 44′ 31″ S	77° 53′ 18″ W	1,623
Ancash	Huaylas	Caraz	8° 58′ 59″ S	77° 49′ 18″ W	2,235
Ancash	Huaylas	Yungay	9° 10′ 37″ S	77° 44′ 48″ W	2,476
Ancash	Huaylas	Carhuaz	9° 19′ 28″ S	77° 35′ 57″ W	2,782
Ancash	Huaylas	Huaraz	9° 31′ 10″ S	77° 32′ 14″ W	3,018
Ancash	Conchucos	Sihuas	8° 33′ 15″ S	77° 37′ 51″ W	2,682
Ancash	Conchucos	Huayllan	8° 49′ 54″ S	77° 26′ 19″ W	3,143
Ancash	Conchucos	Lucma	8° 55′ 59″ S	77° 25′ 11″ W	3,941
Ancash	Conchucos	Masin	9° 21′ 59″ S	77° 05′ 48″ W	2,537
Ancash	Conchucos	Chingas	9° 07′ 04″ S	76° 59′ 25″ W	2,841
Ancash	Conchucos	San Marcos	9° 34′ 03″ S	77° 10′ 41″ W	3,141
Amazonas	Bongara	Janzan	5° 56′ 57″ S	77° 57′ 25″ W	2,630
Piura	Huancabamba	Sondorillo	5° 21′ 05″ S	79° 26′ 48″ W	2,007
Cajamarca	Jaen	San Jose del Alto	5° 25′ 43″ S	79° 07′ 6″ W	1,565
Lima	Huarochiri	Huinco	11° 45′ 54″ S	76° 35′ 41″ W	2,930
Huancavelica	Huaytara	Santo Domingo de Capillas	13° 41′ 27″ S	75° 14′ 17″ W	2,982

**Table 4:** The 162 variable nucleotide positions of the 667-bp cytochrome c oxidase sequence

Collection			Nucleotide position																																																																																																																																																												
Series	Location	Sequence name	22	25	34	38	40	43	46	49	50	58	67	70	79	82	85	91	92	103	106	109	115	121	127	130	133	139	145	154	157	160	163	166	181	184	193	197	199	202	205	206	208	211	212	214	217	220	226	229	232	235	238	253	256	263	266	268	271	274	277	283	284	286	287	289	295	298	301	307	313	316	322	325	328	334	337	343	346	349	352	355	361	362	364	369	370	373	376	379	382	386	388	391	397	400	401	403	407	415	418	421	424	428	430	433	436	442	451	466	475	478	481	482	484	485	490	496	499	500	508	514	517	520	523	526	529	532	533	535	536	538	539	541	547	550	553	556	562	565	568	574	578	580	586	589	595	596	601	604	607	610	613	616	619	622	628	631	634
*Verrucarum*	Amazonas	Ama02	**A**	**C**	**T**	**G**	**A**	**A**	**T**	**T**	**T**	**T**	**A**	**T**	**G**	**T**	**C**	**T**	**T**	**T**	**T**	**A**	**T**	**A**	**T**	**A**	**T**	**T**	**T**	**T**	**T**	**A**	**A**	**A**	**A**	**A**	**C**	**C**	**T**	**T**	**T**	**T**	**A**	**A**	**C**	**T**	**A**	**C**	**T**	**A**	**A**	**T**	**T**	**A**	**C**	**T**	**C**	**T**	**T**	**T**	**T**	**C**	**C**	**T**	**C**	**C**	**A**	**C**	**A**	**A**	**T**	**A**	**A**	**T**	**T**	**T**	**T**	**C**	**T**	**A**	**T**	**T**	**T**	**G**	**T**	**G**	**A**	**A**	**T**	**A**	**T**	**C**	**T**	**A**	**C**	**T**	**T**	**A**	**T**	**A**	**T**	**T**	**A**	**C**	**T**	**A**	**T**	**C**	**A**	**A**	**A**	**G**	**T**	**A**	**T**	**T**	**T**	**A**	**C**	**T**	**T**	**T**	**T**	**A**	**T**	**A**	**T**	**A**	**T**	**A**	**T**	**A**	**C**	**A**	**T**	**T**	**T**	**T**	**A**	**A**	**T**	**A**	**C**	**T**	**T**	**T**	**T**	**T**	**T**	**C**	**A**	**C**	**T**	**C**	**T**	**A**	**A**	**A**	**C**
*Verrucarum*	Amazonas	Ama06	**·**	**·**	**·**	**·**	**·**	**·**	**·**	**·**	**·**	**·**	**·**	**·**	**·**	**·**	**·**	**·**	**·**	**·**	**·**	**·**	**·**	**·**	**·**	**·**	**·**	**·**	**·**	**·**	**·**	**·**	**·**	**·**	**·**	**·**	**·**	**·**	**·**	**·**	**·**	**·**	**·**	**·**	**·**	**·**	**·**	**·**	**·**	**·**	**·**	**·**	**·**	**·**	**·**	**·**	**·**	**·**	**·**	**·**	**·**	**·**	**·**	**·**	**·**	**·**	**·**	**·**	**·**	**·**	**·**	**G**	**·**	**·**	**·**	**·**	**·**	**·**	**·**	**·**	**·**	**·**	**·**	**·**	**·**	**·**	**·**	**·**	**·**	**G**	**·**	**·**	**·**	**·**	**·**	**·**	**·**	**·**	**·**	**·**	**·**	**·**	**·**	**·**	**·**	**·**	**·**	**·**	**·**	**·**	**·**	**·**	**·**	**·**	**·**	**·**	**·**	**·**	**·**	**·**	**·**	**·**	**·**	**·**	**·**	**·**	**·**	**·**	**·**	**·**	**·**	**·**	**·**	**·**	**·**	**·**	**·**	**·**	**·**	**·**	**·**	**·**	**·**	**·**	**·**	**·**	**·**	**·**	**·**	**·**	**·**	**·**	**·**	**·**	**·**	**·**	**·**	**·**	**·**
*Verrucarum*	Amazonas	Ama10	**·**	**·**	**·**	**·**	**·**	**·**	**·**	**·**	**·**	**·**	**·**	**·**	**T**	**·**	**·**	**·**	**·**	**·**	**·**	**·**	**·**	**·**	**·**	**·**	**·**	**·**	**·**	**·**	**·**	**·**	**·**	**·**	**·**	**·**	**·**	**·**	**·**	**·**	**·**	**·**	**·**	**·**	**·**	**·**	**·**	**·**	**·**	**·**	**·**	**·**	**·**	**·**	**·**	**·**	**·**	**·**	**·**	**·**	**·**	**·**	**·**	**·**	**·**	**·**	**·**	**·**	**·**	**·**	**·**	**·**	**·**	**·**	**·**	**·**	**·**	**·**	**·**	**·**	**·**	**·**	**·**	**·**	**·**	**·**	**·**	**·**	**·**	**G**	**·**	**·**	**·**	**·**	**·**	**·**	**·**	**·**	**·**	**·**	**·**	**·**	**·**	**·**	**·**	**·**	**·**	**·**	**·**	**·**	**·**	**·**	**·**	**·**	**·**	**·**	**·**	**·**	**·**	**·**	**·**	**·**	**·**	**·**	**·**	**·**	**·**	**·**	**·**	**·**	**·**	**·**	**·**	**·**	**·**	**·**	**·**	**·**	**·**	**·**	**·**	**·**	**·**	**·**	**·**	**·**	**·**	**·**	**·**	**·**	**·**	**·**	**·**	**·**	**·**	**·**	**·**	**·**	**·**
*Verrucarum*	Amazonas	Ama11	**·**	**·**	**·**	**·**	**·**	**·**	**·**	**·**	**·**	**·**	**·**	**·**	**·**	**·**	**·**	**·**	**·**	**·**	**·**	**·**	**·**	**·**	**·**	**·**	**·**	**·**	**·**	**·**	**·**	**·**	**·**	**·**	**·**	**·**	**·**	**·**	**·**	**·**	**·**	**·**	**·**	**·**	**·**	**·**	**·**	**·**	**·**	**·**	**·**	**·**	**·**	**·**	**·**	**·**	**·**	**·**	**·**	**·**	**·**	**·**	**·**	**·**	**·**	**·**	**·**	**·**	**·**	**·**	**·**	**·**	**·**	**·**	**·**	**·**	**·**	**·**	**·**	**·**	**·**	**·**	**·**	**·**	**·**	**·**	**·**	**·**	**·**	**G**	**·**	**·**	**·**	**·**	**·**	**·**	**·**	**·**	**·**	**·**	**·**	**·**	**·**	**·**	**·**	**·**	**·**	**·**	**·**	**·**	**·**	**·**	**·**	**·**	**·**	**·**	**·**	**·**	**·**	**·**	**·**	**·**	**·**	**·**	**·**	**·**	**·**	**·**	**·**	**·**	**·**	**·**	**·**	**·**	**·**	**·**	**·**	**·**	**·**	**·**	**·**	**·**	**·**	**·**	**·**	**·**	**·**	**·**	**·**	**·**	**·**	**·**	**·**	**·**	**·**	**·**	**·**	**·**	**·**
*Verrucarum*	Amazonas	Ama14	**·**	**·**	**·**	**·**	**·**	**·**	**·**	**·**	**·**	**·**	**·**	**·**	**·**	**·**	**·**	**·**	**·**	**·**	**·**	**·**	**·**	**·**	**·**	**·**	**·**	**·**	**·**	**·**	**·**	**·**	**·**	**·**	**G**	**·**	**·**	**·**	**·**	**·**	**·**	**·**	**·**	**·**	**·**	**·**	**·**	**·**	**·**	**·**	**·**	**·**	**·**	**·**	**·**	**·**	**·**	**·**	**·**	**·**	**·**	**·**	**·**	**·**	**·**	**·**	**·**	**·**	**·**	**·**	**·**	**·**	**·**	**·**	**·**	**·**	**·**	**·**	**·**	**·**	**·**	**·**	**·**	**·**	**·**	**·**	**·**	**·**	**·**	**G**	**·**	**·**	**·**	**·**	**·**	**·**	**·**	**·**	**·**	**·**	**·**	**·**	**·**	**·**	**·**	**·**	**·**	**·**	**·**	**·**	**·**	**·**	**·**	**·**	**·**	**·**	**·**	**·**	**·**	**·**	**·**	**·**	**·**	**·**	**·**	**·**	**·**	**·**	**·**	**·**	**·**	**·**	**·**	**·**	**·**	**·**	**·**	**·**	**·**	**·**	**·**	**·**	**·**	**·**	**·**	**·**	**·**	**·**	**·**	**·**	**·**	**·**	**·**	**·**	**·**	**·**	**·**	**·**	**·**
*Verrucarum*	Cajamarca	Caj01	**G**	**·**	**·**	**·**	**·**	**G**	**·**	**·**	**C**	**C**	**·**	**·**	**T**	**C**	**·**	**·**	**·**	**·**	**·**	**·**	**·**	**G**	**·**	**·**	**·**	**·**	**·**	**C**	**·**	**·**	**T**	**·**	**·**	**·**	**·**	**·**	**·**	**·**	**C**	**·**	**·**	**G**	**·**	**·**	**·**	**T**	**·**	**·**	**·**	**·**	**·**	**·**	**T**	**·**	**·**	**·**	**·**	**·**	**·**	**T**	**·**	**·**	**·**	**·**	**·**	**T**	**·**	**·**	**·**	**·**	**·**	**·**	**·**	**·**	**·**	**·**	**·**	**·**	**·**	**·**	**C**	**·**	**·**	**·**	**·**	**·**	**·**	**T**	**·**	**·**	**·**	**·**	**·**	**·**	**·**	**·**	**·**	**·**	**·**	**·**	**·**	**·**	**·**	**G**	**·**	**·**	**·**	**·**	**·**	**·**	**·**	**·**	**·**	**·**	**·**	**·**	**·**	**·**	**·**	**·**	**·**	**·**	**·**	**·**	**·**	**·**	**·**	**·**	**·**	**·**	**·**	**·**	**·**	**C**	**·**	**·**	**·**	**·**	**·**	**·**	**·**	**·**	**·**	**·**	**·**	**·**	**·**	**·**	**·**	**·**	**·**	**·**	**·**	**·**	**·**	**·**	**·**
*Verrucarum*	Cajamarca	Caj07	**G**	**·**	**C**	**·**	**·**	**G**	**·**	**·**	**C**	**C**	**·**	**·**	**T**	**C**	**·**	**·**	**·**	**·**	**·**	**·**	**·**	**G**	**·**	**·**	**·**	**·**	**·**	**C**	**·**	**·**	**T**	**·**	**·**	**·**	**·**	**·**	**·**	**·**	**C**	**·**	**·**	**G**	**·**	**·**	**·**	**T**	**·**	**·**	**·**	**·**	**·**	**·**	**T**	**·**	**·**	**·**	**·**	**·**	**·**	**T**	**·**	**·**	**·**	**·**	**·**	**T**	**·**	**·**	**·**	**·**	**·**	**·**	**·**	**·**	**·**	**·**	**·**	**·**	**·**	**·**	**C**	**·**	**·**	**·**	**·**	**·**	**·**	**T**	**·**	**·**	**·**	**·**	**·**	**·**	**·**	**·**	**·**	**·**	**·**	**·**	**·**	**·**	**·**	**G**	**·**	**·**	**·**	**·**	**·**	**·**	**·**	**·**	**·**	**·**	**·**	**·**	**·**	**·**	**·**	**·**	**·**	**·**	**·**	**·**	**·**	**·**	**·**	**·**	**·**	**·**	**·**	**·**	**·**	**C**	**·**	**·**	**·**	**·**	**·**	**·**	**·**	**·**	**·**	**·**	**·**	**·**	**·**	**·**	**·**	**·**	**·**	**·**	**·**	**·**	**·**	**·**	**·**
*Verrucarum*	Cajamarca	Caj09	**G**	**·**	**·**	**·**	**·**	**·**	**·**	**·**	**C**	**C**	**·**	**·**	**T**	**C**	**·**	**·**	**·**	**·**	**·**	**·**	**·**	**G**	**·**	**·**	**·**	**·**	**·**	**C**	**·**	**·**	**T**	**·**	**·**	**·**	**·**	**·**	**·**	**·**	**C**	**·**	**·**	**G**	**·**	**·**	**·**	**T**	**·**	**·**	**·**	**·**	**·**	**·**	**T**	**·**	**·**	**·**	**·**	**·**	**·**	**T**	**·**	**·**	**·**	**·**	**·**	**T**	**·**	**·**	**·**	**·**	**·**	**·**	**·**	**·**	**·**	**·**	**·**	**·**	**·**	**·**	**C**	**·**	**·**	**·**	**·**	**·**	**·**	**T**	**·**	**·**	**·**	**·**	**·**	**·**	**·**	**·**	**·**	**·**	**·**	**·**	**·**	**·**	**·**	**G**	**·**	**·**	**·**	**·**	**·**	**·**	**·**	**·**	**·**	**·**	**·**	**·**	**·**	**·**	**·**	**·**	**·**	**·**	**·**	**·**	**·**	**·**	**·**	**·**	**·**	**·**	**·**	**·**	**·**	**C**	**·**	**·**	**·**	**·**	**·**	**·**	**·**	**·**	**·**	**·**	**·**	**·**	**·**	**·**	**·**	**·**	**·**	**·**	**·**	**·**	**·**	**·**	**·**
*Verrucarum*	Cajamarca	Caja05	**G**	**·**	**·**	**·**	**·**	**G**	**·**	**·**	**C**	**C**	**·**	**·**	**T**	**C**	**·**	**·**	**·**	**·**	**·**	**·**	**·**	**G**	**·**	**·**	**·**	**·**	**·**	**C**	**·**	**·**	**T**	**·**	**·**	**·**	**·**	**·**	**·**	**·**	**C**	**·**	**·**	**G**	**·**	**·**	**·**	**T**	**·**	**·**	**·**	**·**	**·**	**·**	**T**	**·**	**·**	**·**	**·**	**·**	**·**	**T**	**·**	**·**	**·**	**·**	**·**	**T**	**·**	**·**	**·**	**G**	**·**	**·**	**·**	**·**	**·**	**·**	**·**	**·**	**·**	**·**	**C**	**·**	**·**	**·**	**·**	**·**	**·**	**T**	**·**	**·**	**·**	**·**	**·**	**·**	**·**	**·**	**·**	**·**	**·**	**·**	**·**	**·**	**·**	**G**	**·**	**·**	**·**	**·**	**·**	**·**	**·**	**·**	**·**	**·**	**·**	**·**	**·**	**·**	**·**	**·**	**·**	**·**	**·**	**·**	**·**	**·**	**·**	**·**	**·**	**·**	**·**	**·**	**·**	**C**	**·**	**·**	**·**	**·**	**·**	**·**	**·**	**·**	**·**	**·**	**·**	**·**	**·**	**·**	**·**	**·**	**·**	**·**	**·**	**·**	**·**	**·**	**·**
*Verrucarum*	Piura	Piu02	**·**	**·**	**·**	**·**	**·**	**G**	**C**	**·**	**·**	**·**	**·**	**·**	**T**	**·**	**·**	**·**	**·**	**·**	**·**	**·**	**·**	**·**	**·**	**·**	**·**	**·**	**·**	**C**	**·**	**·**	**T**	**·**	**·**	**·**	**·**	**·**	**·**	**·**	**C**	**·**	**·**	**G**	**·**	**·**	**·**	**·**	**·**	**·**	**·**	**C**	**·**	**·**	**T**	**·**	**·**	**·**	**·**	**·**	**·**	**T**	**·**	**·**	**·**	**·**	**·**	**·**	**·**	**·**	**·**	**·**	**G**	**·**	**C**	**·**	**·**	**·**	**·**	**·**	**C**	**·**	**·**	**·**	**·**	**·**	**·**	**·**	**·**	**T**	**·**	**·**	**·**	**·**	**·**	**·**	**·**	**·**	**·**	**·**	**·**	**·**	**·**	**·**	**·**	**·**	**·**	**·**	**·**	**·**	**·**	**A**	**·**	**·**	**·**	**·**	**·**	**·**	**·**	**·**	**·**	**·**	**·**	**·**	**·**	**·**	**·**	**·**	**·**	**·**	**·**	**·**	**·**	**·**	**·**	**C**	**·**	**·**	**·**	**·**	**·**	**·**	**·**	**·**	**·**	**·**	**·**	**·**	**·**	**·**	**·**	**·**	**·**	**·**	**·**	**·**	**·**	**·**	**·**
*Verrucarum*	Piura	Piu17	**·**	**·**	**·**	**·**	**·**	**G**	**C**	**·**	**·**	**·**	**·**	**·**	**T**	**·**	**·**	**·**	**·**	**·**	**·**	**·**	**·**	**·**	**·**	**·**	**·**	**·**	**·**	**C**	**·**	**·**	**T**	**·**	**·**	**·**	**·**	**·**	**·**	**·**	**C**	**·**	**·**	**G**	**·**	**·**	**·**	**·**	**·**	**·**	**·**	**C**	**·**	**·**	**T**	**·**	**·**	**·**	**·**	**·**	**·**	**T**	**·**	**·**	**·**	**·**	**·**	**·**	**·**	**·**	**·**	**·**	**G**	**·**	**C**	**·**	**·**	**·**	**·**	**·**	**·**	**·**	**·**	**·**	**·**	**·**	**·**	**·**	**·**	**T**	**·**	**·**	**·**	**·**	**·**	**·**	**·**	**·**	**·**	**·**	**·**	**·**	**·**	**·**	**·**	**·**	**·**	**·**	**·**	**·**	**·**	**A**	**·**	**·**	**·**	**·**	**·**	**·**	**·**	**·**	**·**	**·**	**·**	**·**	**·**	**·**	**·**	**·**	**·**	**·**	**·**	**·**	**·**	**·**	**·**	**C**	**·**	**·**	**·**	**·**	**·**	**·**	**·**	**·**	**·**	**·**	**·**	**·**	**·**	**·**	**·**	**·**	**·**	**·**	**·**	**·**	**·**	**·**	**·**
*Verrucarum*	Piura	Piu22	**·**	**·**	**·**	**·**	**·**	**·**	**C**	**·**	**·**	**·**	**·**	**·**	**T**	**·**	**·**	**·**	**·**	**·**	**·**	**·**	**·**	**·**	**·**	**·**	**·**	**·**	**·**	**C**	**·**	**·**	**T**	**·**	**·**	**·**	**·**	**·**	**·**	**·**	**C**	**·**	**·**	**G**	**·**	**·**	**·**	**·**	**·**	**·**	**·**	**·**	**·**	**·**	**T**	**·**	**·**	**·**	**·**	**·**	**·**	**T**	**·**	**·**	**·**	**·**	**·**	**·**	**·**	**·**	**·**	**G**	**G**	**·**	**·**	**·**	**·**	**T**	**·**	**·**	**·**	**·**	**·**	**·**	**C**	**·**	**·**	**·**	**·**	**T**	**·**	**·**	**·**	**·**	**·**	**·**	**·**	**·**	**·**	**·**	**·**	**·**	**·**	**·**	**·**	**·**	**·**	**·**	**·**	**·**	**·**	**·**	**·**	**·**	**·**	**·**	**·**	**·**	**·**	**·**	**·**	**·**	**·**	**·**	**·**	**·**	**·**	**·**	**·**	**·**	**·**	**·**	**·**	**·**	**·**	**C**	**·**	**·**	**·**	**·**	**·**	**·**	**·**	**·**	**·**	**·**	**·**	**·**	**·**	**·**	**·**	**·**	**·**	**·**	**·**	**·**	**·**	**·**	**·**
*Verrucarum*	Conchucos	Hnco02	**G**	**·**	**C**	**·**	**·**	**G**	**·**	**·**	**C**	**·**	**·**	**·**	**T**	**·**	**·**	**·**	**·**	**·**	**·**	**·**	**·**	**·**	**·**	**·**	**·**	**·**	**·**	**C**	**·**	**·**	**T**	**·**	**·**	**G**	**·**	**·**	**·**	**·**	**·**	**·**	**·**	**G**	**·**	**·**	**·**	**·**	**·**	**·**	**·**	**·**	**·**	**·**	**T**	**·**	**·**	**·**	**·**	**·**	**·**	**T**	**·**	**·**	**·**	**·**	**·**	**·**	**·**	**·**	**·**	**G**	**·**	**·**	**·**	**·**	**·**	**·**	**·**	**·**	**·**	**·**	**C**	**·**	**·**	**·**	**·**	**·**	**·**	**T**	**·**	**·**	**·**	**·**	**·**	**·**	**·**	**·**	**·**	**·**	**·**	**·**	**·**	**·**	**·**	**·**	**·**	**·**	**·**	**·**	**·**	**A**	**·**	**·**	**·**	**·**	**·**	**·**	**·**	**·**	**·**	**·**	**·**	**·**	**·**	**·**	**·**	**·**	**·**	**·**	**·**	**·**	**·**	**·**	**C**	**·**	**·**	**·**	**·**	**·**	**·**	**·**	**·**	**·**	**·**	**·**	**·**	**·**	**·**	**·**	**·**	**·**	**·**	**·**	**·**	**·**	**G**	**G**	**T**
*Verrucarum*	Conchucos	Sihua07	**·**	**·**	**·**	**·**	**·**	**G**	**·**	**·**	**C**	**·**	**·**	**·**	**T**	**·**	**·**	**·**	**·**	**·**	**·**	**·**	**·**	**·**	**·**	**·**	**·**	**·**	**·**	**C**	**·**	**·**	**T**	**·**	**·**	**G**	**·**	**·**	**·**	**·**	**·**	**·**	**·**	**G**	**·**	**·**	**·**	**·**	**·**	**·**	**·**	**·**	**·**	**·**	**T**	**·**	**·**	**·**	**·**	**·**	**·**	**T**	**·**	**·**	**·**	**·**	**·**	**·**	**·**	**·**	**·**	**·**	**·**	**·**	**·**	**·**	**·**	**·**	**·**	**·**	**·**	**·**	**C**	**A**	**·**	**·**	**·**	**·**	**·**	**T**	**·**	**·**	**·**	**·**	**·**	**·**	**·**	**·**	**·**	**·**	**·**	**·**	**·**	**·**	**·**	**·**	**·**	**·**	**·**	**·**	**·**	**A**	**·**	**·**	**·**	**·**	**·**	**·**	**·**	**·**	**·**	**·**	**·**	**·**	**·**	**·**	**·**	**·**	**·**	**·**	**·**	**·**	**·**	**·**	**C**	**·**	**·**	**·**	**·**	**·**	**·**	**·**	**·**	**·**	**·**	**·**	**·**	**·**	**·**	**·**	**·**	**·**	**·**	**·**	**·**	**·**	**·**	**·**	**·**
*Verrucarum*	Conchucos	Chngas11	**·**	**·**	**·**	**·**	**·**	**G**	**·**	**·**	**C**	**·**	**·**	**·**	**T**	**·**	**·**	**·**	**·**	**·**	**·**	**·**	**·**	**·**	**·**	**·**	**·**	**·**	**·**	**C**	**·**	**·**	**T**	**·**	**·**	**G**	**·**	**·**	**·**	**·**	**·**	**·**	**·**	**G**	**·**	**·**	**·**	**·**	**·**	**·**	**·**	**·**	**·**	**·**	**T**	**·**	**·**	**·**	**·**	**·**	**·**	**T**	**·**	**·**	**·**	**·**	**·**	**·**	**·**	**·**	**·**	**·**	**·**	**·**	**·**	**·**	**·**	**·**	**·**	**·**	**·**	**·**	**C**	**·**	**·**	**·**	**·**	**·**	**·**	**T**	**·**	**·**	**·**	**·**	**·**	**·**	**·**	**·**	**·**	**·**	**·**	**·**	**·**	**·**	**·**	**·**	**·**	**·**	**·**	**·**	**·**	**A**	**·**	**·**	**·**	**·**	**·**	**·**	**·**	**·**	**·**	**·**	**·**	**·**	**·**	**·**	**·**	**·**	**·**	**·**	**·**	**·**	**·**	**·**	**·**	**·**	**·**	**·**	**·**	**·**	**·**	**·**	**·**	**·**	**·**	**·**	**·**	**·**	**·**	**·**	**·**	**·**	**·**	**·**	**·**	**·**	**G**	**G**	**T**
*Verrucarum*	Conchucos	Masn17	**·**	**·**	**·**	**·**	**·**	**G**	**·**	**·**	**C**	**·**	**·**	**·**	**T**	**·**	**·**	**·**	**·**	**·**	**·**	**·**	**·**	**·**	**·**	**·**	**·**	**·**	**·**	**C**	**·**	**·**	**T**	**·**	**·**	**G**	**·**	**·**	**·**	**·**	**·**	**·**	**·**	**G**	**·**	**·**	**·**	**·**	**·**	**·**	**·**	**·**	**·**	**·**	**T**	**·**	**·**	**·**	**·**	**·**	**·**	**T**	**·**	**·**	**·**	**·**	**·**	**·**	**·**	**·**	**·**	**G**	**·**	**·**	**·**	**·**	**·**	**·**	**·**	**·**	**·**	**·**	**C**	**·**	**·**	**·**	**·**	**·**	**·**	**T**	**·**	**·**	**·**	**·**	**·**	**·**	**·**	**·**	**·**	**·**	**·**	**·**	**·**	**·**	**·**	**·**	**·**	**·**	**·**	**·**	**·**	**A**	**·**	**·**	**·**	**·**	**·**	**·**	**·**	**·**	**·**	**·**	**·**	**·**	**·**	**·**	**·**	**·**	**·**	**·**	**·**	**·**	**·**	**·**	**·**	**·**	**·**	**·**	**·**	**·**	**·**	**·**	**·**	**·**	**·**	**·**	**·**	**·**	**·**	**·**	**·**	**·**	**·**	**·**	**·**	**·**	**G**	**G**	**T**
*Verrucarum*	Conchucos	Lverru	**G**	**·**	**C**	**·**	**·**	**G**	**·**	**·**	**C**	**·**	**·**	**·**	**T**	**·**	**·**	**·**	**·**	**·**	**·**	**·**	**·**	**·**	**·**	**·**	**·**	**·**	**·**	**C**	**·**	**·**	**T**	**·**	**·**	**G**	**·**	**·**	**·**	**·**	**·**	**·**	**·**	**G**	**·**	**·**	**·**	**·**	**·**	**·**	**·**	**·**	**·**	**·**	**T**	**·**	**·**	**·**	**·**	**·**	**·**	**T**	**·**	**·**	**·**	**·**	**·**	**·**	**·**	**·**	**·**	**·**	**·**	**·**	**·**	**·**	**·**	**·**	**·**	**·**	**·**	**·**	**C**	**·**	**·**	**·**	**·**	**·**	**·**	**T**	**·**	**·**	**·**	**·**	**·**	**·**	**·**	**·**	**·**	**·**	**·**	**·**	**·**	**·**	**·**	**·**	**·**	**·**	**·**	**·**	**·**	**A**	**·**	**·**	**·**	**·**	**·**	**·**	**·**	**·**	**·**	**·**	**·**	**·**	**·**	**·**	**·**	**·**	**·**	**·**	**·**	**·**	**·**	**·**	**C**	**C**	**·**	**·**	**·**	**·**	**·**	**·**	**·**	**·**	**·**	**·**	**·**	**·**	**·**	**·**	**·**	**·**	**·**	**·**	**·**	**G**	**G**	**G**	**T**
*Verrucarum*	Conchucos	Lverru2	**G**	**·**	**C**	**·**	**·**	**G**	**·**	**·**	**C**	**·**	**·**	**·**	**T**	**·**	**·**	**·**	**·**	**·**	**·**	**·**	**·**	**·**	**·**	**·**	**·**	**·**	**·**	**C**	**·**	**·**	**T**	**·**	**·**	**G**	**·**	**·**	**·**	**·**	**·**	**·**	**·**	**G**	**·**	**·**	**·**	**·**	**·**	**·**	**·**	**·**	**·**	**·**	**T**	**·**	**·**	**·**	**·**	**·**	**·**	**T**	**·**	**·**	**·**	**·**	**·**	**·**	**·**	**·**	**·**	**·**	**·**	**·**	**·**	**·**	**·**	**·**	**·**	**·**	**·**	**·**	**C**	**·**	**·**	**·**	**·**	**·**	**·**	**T**	**·**	**·**	**·**	**·**	**·**	**·**	**·**	**·**	**·**	**·**	**·**	**·**	**·**	**·**	**·**	**·**	**·**	**·**	**·**	**·**	**·**	**A**	**·**	**·**	**·**	**·**	**·**	**·**	**·**	**·**	**·**	**·**	**·**	**·**	**·**	**·**	**·**	**·**	**·**	**·**	**·**	**·**	**·**	**·**	**C**	**·**	**·**	**·**	**·**	**·**	**·**	**·**	**·**	**·**	**·**	**·**	**·**	**·**	**·**	**·**	**·**	**·**	**·**	**·**	**·**	**·**	**G**	**G**	**T**
*Verrucarum*	Purisima	Casma01	**·**	**·**	**G**	**·**	**·**	**G**	**·**	**·**	**·**	**·**	**·**	**·**	**C**	**·**	**·**	**·**	**·**	**·**	**·**	**·**	**·**	**·**	**·**	**·**	**·**	**·**	**·**	**·**	**·**	**·**	**·**	**·**	**·**	**·**	**·**	**·**	**·**	**·**	**·**	**·**	**·**	**G**	**·**	**·**	**·**	**·**	**·**	**·**	**·**	**·**	**·**	**·**	**T**	**·**	**·**	**·**	**·**	**·**	**·**	**T**	**·**	**·**	**·**	**·**	**·**	**T**	**·**	**·**	**·**	**G**	**·**	**·**	**·**	**·**	**·**	**·**	**·**	**·**	**·**	**·**	**·**	**·**	**C**	**·**	**·**	**·**	**·**	**T**	**·**	**·**	**·**	**·**	**·**	**·**	**·**	**·**	**·**	**·**	**·**	**·**	**·**	**·**	**·**	**·**	**·**	**·**	**·**	**·**	**·**	**·**	**·**	**·**	**·**	**·**	**·**	**·**	**·**	**·**	**·**	**·**	**·**	**G**	**·**	**·**	**·**	**·**	**·**	**·**	**·**	**·**	**·**	**·**	**·**	**·**	**·**	**·**	**·**	**·**	**·**	**·**	**·**	**·**	**·**	**·**	**·**	**·**	**·**	**·**	**·**	**·**	**·**	**T**	**·**	**G**	**G**	**·**	**·**
*Verrucarum*	Purisima	Yumpe15	**·**	**·**	**A**	**·**	**·**	**G**	**·**	**·**	**·**	**·**	**·**	**·**	**C**	**·**	**·**	**·**	**·**	**·**	**·**	**·**	**·**	**·**	**·**	**·**	**·**	**·**	**·**	**·**	**·**	**·**	**·**	**·**	**·**	**·**	**·**	**·**	**·**	**·**	**·**	**·**	**·**	**G**	**·**	**·**	**·**	**·**	**·**	**·**	**·**	**·**	**·**	**·**	**T**	**·**	**·**	**·**	**·**	**·**	**·**	**T**	**·**	**·**	**·**	**·**	**·**	**T**	**·**	**·**	**·**	**·**	**·**	**·**	**·**	**·**	**·**	**·**	**·**	**·**	**·**	**·**	**·**	**·**	**C**	**·**	**·**	**·**	**·**	**T**	**·**	**·**	**·**	**·**	**·**	**·**	**·**	**·**	**·**	**·**	**·**	**·**	**·**	**·**	**·**	**·**	**·**	**·**	**·**	**·**	**·**	**·**	**·**	**·**	**·**	**·**	**·**	**·**	**·**	**·**	**·**	**·**	**·**	**G**	**·**	**·**	**·**	**·**	**·**	**·**	**·**	**·**	**·**	**·**	**·**	**·**	**·**	**·**	**·**	**·**	**·**	**·**	**·**	**·**	**·**	**·**	**·**	**·**	**·**	**·**	**·**	**·**	**·**	**T**	**·**	**G**	**G**	**·**	**·**
*Verrucarum*	Purisima	Yuram01	**·**	**·**	**·**	**·**	**·**	**G**	**·**	**·**	**·**	**·**	**·**	**·**	**T**	**·**	**·**	**·**	**·**	**·**	**·**	**·**	**·**	**·**	**·**	**·**	**·**	**·**	**·**	**·**	**·**	**·**	**·**	**·**	**·**	**·**	**·**	**·**	**·**	**·**	**·**	**·**	**·**	**G**	**·**	**·**	**·**	**·**	**·**	**·**	**·**	**·**	**·**	**·**	**T**	**·**	**·**	**·**	**C**	**·**	**·**	**T**	**·**	**·**	**·**	**·**	**·**	**T**	**·**	**·**	**·**	**·**	**·**	**·**	**·**	**·**	**·**	**·**	**·**	**·**	**·**	**·**	**·**	**·**	**C**	**·**	**·**	**·**	**·**	**T**	**·**	**·**	**·**	**·**	**·**	**·**	**·**	**·**	**·**	**·**	**·**	**·**	**·**	**·**	**·**	**·**	**·**	**·**	**·**	**·**	**·**	**·**	**·**	**·**	**·**	**·**	**·**	**·**	**·**	**·**	**·**	**·**	**·**	**·**	**·**	**·**	**·**	**·**	**·**	**·**	**·**	**·**	**·**	**·**	**·**	**C**	**·**	**·**	**·**	**·**	**·**	**·**	**·**	**·**	**·**	**·**	**·**	**·**	**·**	**·**	**·**	**·**	**·**	**·**	**·**	**G**	**G**	**·**	**·**
*Verrucarum*	Purisima	Yuram02	**·**	**·**	**·**	**·**	**·**	**G**	**·**	**·**	**·**	**·**	**·**	**·**	**T**	**·**	**·**	**·**	**·**	**·**	**·**	**·**	**·**	**·**	**·**	**·**	**·**	**·**	**·**	**·**	**·**	**·**	**·**	**·**	**·**	**·**	**·**	**·**	**·**	**·**	**·**	**·**	**·**	**G**	**·**	**·**	**·**	**·**	**·**	**·**	**·**	**·**	**·**	**·**	**T**	**·**	**·**	**·**	**C**	**·**	**·**	**·**	**·**	**·**	**·**	**·**	**·**	**T**	**·**	**·**	**·**	**·**	**·**	**·**	**·**	**·**	**·**	**·**	**·**	**·**	**·**	**·**	**·**	**·**	**C**	**·**	**·**	**·**	**·**	**T**	**·**	**·**	**·**	**·**	**·**	**·**	**·**	**·**	**·**	**·**	**·**	**·**	**·**	**·**	**·**	**·**	**·**	**·**	**·**	**·**	**·**	**·**	**·**	**·**	**·**	**·**	**·**	**G**	**·**	**·**	**·**	**·**	**·**	**·**	**C**	**·**	**·**	**·**	**·**	**·**	**·**	**·**	**·**	**·**	**·**	**C**	**·**	**·**	**·**	**·**	**·**	**·**	**·**	**·**	**·**	**·**	**·**	**·**	**·**	**·**	**·**	**·**	**·**	**·**	**·**	**·**	**G**	**·**	**·**
*Verrucarum*	Purisima	Maya2883	**·**	**·**	**·**	**·**	**·**	**G**	**·**	**·**	**·**	**·**	**·**	**·**	**T**	**·**	**·**	**·**	**·**	**·**	**·**	**·**	**·**	**·**	**·**	**·**	**·**	**·**	**·**	**·**	**·**	**·**	**·**	**·**	**·**	**·**	**·**	**·**	**·**	**·**	**·**	**·**	**·**	**G**	**·**	**·**	**·**	**·**	**·**	**·**	**·**	**·**	**·**	**·**	**T**	**·**	**·**	**·**	**C**	**·**	**·**	**T**	**·**	**·**	**·**	**·**	**·**	**T**	**·**	**·**	**·**	**G**	**·**	**·**	**·**	**·**	**·**	**·**	**·**	**·**	**·**	**·**	**·**	**·**	**C**	**·**	**·**	**·**	**·**	**T**	**·**	**·**	**·**	**·**	**·**	**·**	**·**	**·**	**·**	**·**	**·**	**·**	**·**	**·**	**·**	**·**	**·**	**·**	**·**	**·**	**·**	**·**	**·**	**·**	**·**	**·**	**·**	**·**	**·**	**·**	**·**	**·**	**·**	**·**	**·**	**·**	**·**	**·**	**·**	**·**	**·**	**·**	**·**	**·**	**·**	**C**	**·**	**·**	**·**	**·**	**·**	**·**	**·**	**·**	**·**	**·**	**·**	**·**	**·**	**·**	**·**	**·**	**·**	**·**	**·**	**G**	**G**	**·**	**·**
*Verrucarum*	Purisima	Shan06	**·**	**·**	**·**	**·**	**·**	**G**	**·**	**·**	**·**	**·**	**·**	**·**	**T**	**·**	**·**	**·**	**·**	**·**	**·**	**·**	**·**	**·**	**·**	**·**	**·**	**·**	**·**	**·**	**·**	**·**	**·**	**·**	**·**	**·**	**·**	**·**	**·**	**·**	**·**	**·**	**·**	**G**	**·**	**·**	**·**	**·**	**·**	**·**	**·**	**·**	**·**	**·**	**T**	**·**	**·**	**·**	**C**	**·**	**·**	**T**	**·**	**·**	**·**	**·**	**·**	**T**	**·**	**·**	**·**	**·**	**·**	**·**	**·**	**·**	**·**	**·**	**·**	**·**	**·**	**·**	**·**	**·**	**C**	**·**	**·**	**·**	**·**	**T**	**·**	**·**	**·**	**·**	**·**	**·**	**·**	**·**	**·**	**·**	**·**	**·**	**·**	**·**	**·**	**·**	**·**	**·**	**·**	**·**	**·**	**·**	**·**	**·**	**·**	**·**	**·**	**·**	**·**	**·**	**·**	**·**	**·**	**·**	**·**	**·**	**·**	**·**	**·**	**·**	**·**	**·**	**·**	**·**	**·**	**C**	**·**	**·**	**·**	**·**	**G**	**·**	**·**	**·**	**·**	**·**	**·**	**·**	**·**	**·**	**·**	**·**	**·**	**·**	**·**	**G**	**G**	**·**	**·**
*Verrucarum*	Huancavelica	Huan811	**·**	**·**	**·**	**·**	**·**	**·**	**·**	**·**	**·**	**·**	**·**	**·**	**T**	**·**	**·**	**·**	**·**	**·**	**·**	**·**	**·**	**·**	**·**	**·**	**·**	**·**	**·**	**·**	**·**	**·**	**·**	**·**	**·**	**·**	**·**	**·**	**·**	**·**	**·**	**·**	**·**	**G**	**·**	**C**	**·**	**·**	**·**	**·**	**·**	**·**	**·**	**·**	**T**	**·**	**·**	**·**	**·**	**·**	**·**	**T**	**·**	**·**	**·**	**·**	**·**	**T**	**·**	**·**	**·**	**·**	**·**	**·**	**·**	**·**	**·**	**·**	**·**	**·**	**·**	**·**	**·**	**·**	**C**	**·**	**·**	**·**	**·**	**T**	**·**	**·**	**·**	**·**	**·**	**·**	**·**	**·**	**·**	**·**	**·**	**·**	**·**	**·**	**·**	**·**	**·**	**·**	**·**	**·**	**·**	**·**	**·**	**·**	**·**	**·**	**·**	**G**	**·**	**·**	**·**	**·**	**·**	**·**	**·**	**·**	**·**	**·**	**·**	**·**	**·**	**·**	**·**	**·**	**·**	**·**	**·**	**·**	**·**	**·**	**·**	**·**	**·**	**·**	**·**	**·**	**·**	**·**	**C**	**·**	**·**	**·**	**·**	**T**	**·**	**G**	**G**	**·**	**T**
*Verrucarum*	Lima	Lim04	**·**	**·**	**·**	**·**	**·**	**G**	**·**	**·**	**·**	**·**	**·**	**·**	**T**	**·**	**·**	**·**	**·**	**·**	**·**	**·**	**·**	**·**	**·**	**·**	**·**	**·**	**·**	**·**	**·**	**·**	**·**	**·**	**·**	**·**	**·**	**·**	**·**	**·**	**·**	**·**	**·**	**G**	**·**	**C**	**·**	**·**	**·**	**·**	**·**	**·**	**·**	**·**	**T**	**·**	**·**	**·**	**·**	**·**	**·**	**T**	**·**	**·**	**·**	**·**	**·**	**T**	**·**	**·**	**·**	**G**	**·**	**·**	**·**	**·**	**·**	**·**	**·**	**·**	**·**	**·**	**·**	**·**	**C**	**·**	**·**	**·**	**·**	**T**	**·**	**·**	**·**	**·**	**·**	**·**	**·**	**·**	**·**	**·**	**·**	**·**	**·**	**·**	**·**	**·**	**·**	**·**	**·**	**·**	**·**	**·**	**·**	**·**	**·**	**·**	**·**	**G**	**·**	**·**	**·**	**·**	**·**	**·**	**·**	**·**	**·**	**·**	**·**	**·**	**·**	**·**	**·**	**·**	**·**	**·**	**·**	**·**	**·**	**·**	**·**	**·**	**·**	**·**	**·**	**·**	**·**	**·**	**C**	**·**	**·**	**·**	**·**	**T**	**·**	**G**	**·**	**·**	**T**
*Verrucarum*	Lima	Lim05	**·**	**·**	**·**	**·**	**·**	**G**	**·**	**·**	**·**	**·**	**·**	**·**	**T**	**·**	**·**	**·**	**·**	**·**	**·**	**·**	**·**	**·**	**·**	**·**	**·**	**·**	**·**	**·**	**·**	**·**	**·**	**·**	**·**	**·**	**·**	**·**	**·**	**·**	**·**	**·**	**·**	**G**	**·**	**C**	**·**	**·**	**·**	**·**	**·**	**·**	**·**	**·**	**T**	**·**	**·**	**·**	**·**	**·**	**·**	**T**	**·**	**·**	**·**	**·**	**·**	**T**	**·**	**·**	**·**	**G**	**·**	**·**	**·**	**·**	**·**	**·**	**·**	**·**	**·**	**·**	**·**	**·**	**C**	**·**	**·**	**·**	**·**	**T**	**·**	**·**	**·**	**·**	**·**	**·**	**·**	**·**	**·**	**·**	**·**	**·**	**·**	**·**	**·**	**·**	**·**	**·**	**·**	**·**	**·**	**·**	**·**	**·**	**·**	**·**	**·**	**G**	**·**	**·**	**·**	**·**	**·**	**·**	**·**	**·**	**·**	**·**	**·**	**·**	**·**	**·**	**·**	**·**	**·**	**·**	**·**	**·**	**·**	**·**	**·**	**·**	**·**	**·**	**·**	**·**	**·**	**·**	**C**	**·**	**·**	**·**	**·**	**T**	**·**	**G**	**G**	**·**	**T**
*Verrucarum*	Lima	Lim07	**·**	**·**	**·**	**·**	**·**	**·**	**·**	**·**	**·**	**·**	**·**	**·**	**T**	**·**	**·**	**·**	**·**	**·**	**·**	**·**	**·**	**·**	**·**	**·**	**·**	**·**	**·**	**·**	**·**	**·**	**·**	**·**	**·**	**·**	**·**	**·**	**·**	**·**	**·**	**·**	**·**	**G**	**·**	**C**	**·**	**·**	**·**	**·**	**·**	**·**	**·**	**·**	**T**	**·**	**·**	**·**	**·**	**·**	**·**	**T**	**·**	**·**	**·**	**·**	**·**	**T**	**·**	**·**	**·**	**·**	**·**	**·**	**·**	**·**	**·**	**·**	**·**	**·**	**·**	**·**	**·**	**·**	**C**	**·**	**·**	**·**	**·**	**T**	**·**	**·**	**·**	**·**	**·**	**·**	**·**	**·**	**·**	**·**	**·**	**·**	**·**	**·**	**·**	**·**	**·**	**·**	**·**	**·**	**·**	**·**	**·**	**·**	**·**	**·**	**·**	**G**	**·**	**·**	**·**	**·**	**·**	**·**	**·**	**·**	**·**	**·**	**·**	**·**	**·**	**·**	**·**	**·**	**·**	**·**	**·**	**·**	**·**	**·**	**·**	**·**	**·**	**·**	**·**	**·**	**·**	**·**	**C**	**·**	**·**	**·**	**·**	**T**	**·**	**G**	**·**	**·**	**T**
*Verrucarum*	Lima	Lim17	**·**	**·**	**·**	**·**	**·**	**·**	**·**	**·**	**·**	**·**	**·**	**·**	**T**	**·**	**·**	**·**	**·**	**·**	**·**	**·**	**·**	**·**	**·**	**·**	**·**	**·**	**·**	**·**	**·**	**·**	**·**	**·**	**·**	**·**	**·**	**·**	**·**	**·**	**·**	**·**	**·**	**G**	**·**	**C**	**·**	**·**	**·**	**·**	**·**	**·**	**·**	**·**	**T**	**·**	**·**	**·**	**·**	**·**	**·**	**T**	**·**	**·**	**·**	**·**	**·**	**T**	**·**	**·**	**·**	**·**	**·**	**·**	**·**	**·**	**·**	**·**	**·**	**·**	**·**	**·**	**·**	**·**	**C**	**·**	**·**	**·**	**·**	**T**	**·**	**·**	**·**	**·**	**·**	**·**	**·**	**·**	**·**	**·**	**·**	**·**	**·**	**·**	**·**	**·**	**·**	**·**	**·**	**·**	**·**	**A**	**·**	**·**	**·**	**·**	**·**	**G**	**·**	**·**	**·**	**·**	**·**	**·**	**·**	**·**	**·**	**·**	**·**	**·**	**·**	**·**	**·**	**·**	**·**	**·**	**·**	**·**	**·**	**·**	**·**	**·**	**·**	**·**	**·**	**·**	**·**	**·**	**C**	**·**	**·**	**·**	**·**	**T**	**·**	**G**	**G**	**·**	**T**
*Verrucarum*	Lima	Lim28	**·**	**·**	**·**	**·**	**·**	**G**	**·**	**·**	**·**	**·**	**·**	**·**	**T**	**·**	**·**	**·**	**·**	**·**	**·**	**·**	**·**	**·**	**·**	**·**	**·**	**·**	**·**	**·**	**·**	**·**	**·**	**·**	**·**	**·**	**·**	**·**	**·**	**·**	**·**	**·**	**·**	**G**	**·**	**C**	**·**	**·**	**·**	**·**	**·**	**·**	**·**	**·**	**T**	**·**	**·**	**·**	**·**	**·**	**·**	**T**	**·**	**·**	**·**	**·**	**·**	**T**	**·**	**·**	**·**	**G**	**·**	**·**	**·**	**·**	**·**	**·**	**·**	**·**	**·**	**·**	**·**	**·**	**C**	**·**	**·**	**·**	**·**	**T**	**·**	**·**	**·**	**·**	**·**	**·**	**·**	**·**	**·**	**·**	**·**	**·**	**·**	**·**	**·**	**·**	**·**	**·**	**·**	**·**	**·**	**·**	**·**	**·**	**·**	**·**	**·**	**G**	**·**	**·**	**·**	**·**	**·**	**·**	**·**	**·**	**·**	**·**	**·**	**·**	**·**	**·**	**·**	**·**	**·**	**·**	**·**	**·**	**·**	**·**	**·**	**·**	**·**	**·**	**·**	**·**	**·**	**·**	**C**	**·**	**·**	**·**	**·**	**T**	**·**	**·**	**G**	**·**	**T**
*Verrucarum*	Colombia	Landina	**G**	**·**	**A**	**·**	**C**	**T**	**C**	**·**	**·**	**·**	**·**	**·**	**C**	**C**	**A**	**·**	**·**	**C**	**C**	**·**	**·**	**·**	**·**	**·**	**·**	**·**	**C**	**·**	**·**	**·**	**T**	**·**	**·**	**C**	**·**	**T**	**A**	**C**	**·**	**C**	**·**	**·**	**·**	**·**	**G**	**T**	**·**	**·**	**·**	**C**	**C**	**·**	**T**	**·**	**·**	**·**	**·**	**·**	**·**	**T**	**T**	**A**	**·**	**·**	**·**	**A**	**·**	**T**	**·**	**·**	**G**	**A**	**A**	**·**	**A**	**·**	**A**	**·**	**A**	**·**	**·**	**·**	**C**	**·**	**·**	**·**	**A**	**·**	**·**	**T**	**A**	**·**	**·**	**C**	**C**	**T**	**·**	**G**	**·**	**C**	**·**	**·**	**·**	**·**	**·**	**T**	**T**	**·**	**C**	**A**	**C**	**·**	**C**	**·**	**C**	**·**	**T**	**·**	**C**	**A**	**·**	**·**	**·**	**·**	**A**	**T**	**·**	**·**	**C**	**T**	**·**	**·**	**C**	**A**	**·**	**·**	**·**	**·**	**A**	**T**	**·**	**·**	**·**	**C**	**·**	**·**	**·**	**T**	**·**	**T**	**·**	**·**	**·**	**·**	**·**	**·**	**·**
*Verrucarum*	Colombia	Lnevesi	**·**	**·**	**A**	**·**	**·**	**·**	**C**	**C**	**·**	**·**	**T**	**A**	**T**	**·**	**T**	**·**	**·**	**·**	**C**	**G**	**·**	**·**	**·**	**·**	**A**	**C**	**A**	**·**	**·**	**·**	**T**	**G**	**T**	**·**	**·**	**T**	**A**	**C**	**·**	**·**	**·**	**·**	**·**	**·**	**·**	**·**	**·**	**·**	**·**	**·**	**·**	**·**	**T**	**C**	**T**	**A**	**·**	**·**	**A**	**A**	**·**	**·**	**·**	**T**	**T**	**T**	**T**	**T**	**A**	**·**	**·**	**·**	**A**	**A**	**·**	**·**	**·**	**T**	**·**	**A**	**·**	**·**	**·**	**·**	**T**	**·**	**·**	**T**	**·**	**·**	**·**	**·**	**·**	**A**	**C**	**T**	**C**	**G**	**C**	**·**	**T**	**T**	**A**	**·**	**A**	**T**	**·**	**·**	**·**	**A**	**·**	**·**	**·**	**C**	**C**	**·**	**T**	**·**	**C**	**A**	**A**	**·**	**·**	**T**	**A**	**T**	**·**	**·**	**C**	**T**	**·**	**T**	**·**	**A**	**·**	**G**	**T**	**·**	**·**	**C**	**·**	**·**	**A**	**·**	**·**	**·**	**·**	**A**	**C**	**·**	**·**	**·**	**A**	**·**	**G**	**·**	**·**
*Townsendi*	Colombia	Llongifl	**·**	**·**	**·**	**·**	**·**	**·**	**·**	**A**	**·**	**·**	**·**	**A**	**A**	**C**	**A**	**·**	**·**	**·**	**·**	**·**	**·**	**·**	**·**	**T**	**·**	**·**	**·**	**·**	**C**	**·**	**·**	**·**	**·**	**·**	**T**	**T**	**A**	**·**	**·**	**·**	**·**	**·**	**T**	**A**	**·**	**·**	**·**	**G**	**T**	**C**	**·**	**·**	**A**	**·**	**T**	**A**	**C**	**C**	**·**	**A**	**T**	**A**	**·**	**T**	**·**	**·**	**·**	**T**	**·**	**G**	**G**	**·**	**·**	**A**	**·**	**·**	**·**	**T**	**·**	**·**	**·**	**·**	**·**	**·**	**·**	**·**	**C**	**·**	**C**	**T**	**A**	**·**	**T**	**·**	**·**	**·**	**·**	**T**	**·**	**C**	**T**	**T**	**A**	**G**	**C**	**T**	**T**	**·**	**·**	**A**	**·**	**·**	**·**	**·**	**·**	**·**	**T**	**·**	**A**	**·**	**·**	**T**	**·**	**T**	**·**	**C**	**C**	**T**	**C**	**C**	**T**	**·**	**C**	**G**	**·**	**·**	**T**	**G**	**·**	**T**	**T**	**A**	**·**	**·**	**·**	**·**	**·**	**·**	**C**	**·**	**·**	**·**	**·**	**C**	**G**	**·**	**·**
*Townsendi*	Colombia	Llongifl2	**·**	**·**	**·**	**·**	**·**	**·**	**·**	**A**	**·**	**·**	**·**	**A**	**A**	**C**	**A**	**·**	**·**	**·**	**·**	**·**	**·**	**·**	**·**	**T**	**·**	**·**	**·**	**·**	**C**	**·**	**·**	**·**	**·**	**G**	**T**	**T**	**A**	**·**	**·**	**·**	**·**	**·**	**T**	**A**	**G**	**·**	**·**	**G**	**T**	**C**	**·**	**·**	**A**	**·**	**T**	**A**	**C**	**C**	**·**	**A**	**T**	**A**	**·**	**T**	**·**	**·**	**·**	**T**	**·**	**G**	**·**	**·**	**·**	**A**	**·**	**·**	**·**	**T**	**·**	**·**	**·**	**·**	**·**	**·**	**·**	**·**	**C**	**·**	**·**	**T**	**A**	**·**	**T**	**·**	**·**	**·**	**·**	**T**	**·**	**C**	**T**	**T**	**A**	**G**	**C**	**T**	**T**	**·**	**·**	**A**	**·**	**·**	**·**	**·**	**·**	**·**	**T**	**·**	**G**	**·**	**·**	**T**	**·**	**T**	**·**	**C**	**C**	**T**	**C**	**C**	**T**	**·**	**C**	**G**	**·**	**·**	**T**	**G**	**·**	**T**	**T**	**A**	**·**	**·**	**·**	**·**	**·**	**·**	**C**	**·**	**·**	**·**	**·**	**C**	**G**	**·**	**·**
*Townsendi*	Colombia	Lsauroida	**·**	**·**	**·**	**·**	**·**	**·**	**·**	**A**	**·**	**·**	**·**	**A**	**A**	**·**	**A**	**·**	**·**	**·**	**·**	**·**	**·**	**·**	**·**	**T**	**·**	**·**	**·**	**·**	**C**	**·**	**·**	**·**	**·**	**G**	**T**	**T**	**A**	**·**	**·**	**·**	**·**	**·**	**T**	**A**	**G**	**·**	**·**	**G**	**T**	**C**	**·**	**·**	**A**	**·**	**T**	**A**	**C**	**C**	**·**	**·**	**T**	**A**	**·**	**T**	**·**	**·**	**·**	**T**	**·**	**G**	**G**	**·**	**·**	**A**	**·**	**·**	**·**	**T**	**·**	**·**	**·**	**·**	**·**	**A**	**·**	**·**	**C**	**·**	**C**	**T**	**A**	**·**	**T**	**·**	**·**	**·**	**·**	**T**	**·**	**C**	**T**	**T**	**A**	**G**	**C**	**T**	**T**	**·**	**·**	**A**	**·**	**·**	**·**	**·**	**·**	**·**	**T**	**·**	**A**	**·**	**·**	**T**	**·**	**T**	**·**	**C**	**C**	**T**	**C**	**C**	**T**	**·**	**C**	**G**	**·**	**·**	**T**	**·**	**·**	**T**	**T**	**A**	**·**	**·**	**·**	**·**	**·**	**·**	**C**	**·**	**·**	**·**	**·**	**C**	**G**	**·**	**·**
*Townsendi*	Colombia	Lyoungi	**G**	**·**	**·**	**·**	**·**	**G**	**·**	**A**	**·**	**C**	**·**	**A**	**T**	**C**	**A**	**C**	**·**	**·**	**·**	**·**	**·**	**·**	**G**	**T**	**·**	**·**	**·**	**C**	**C**	**G**	**·**	**·**	**·**	**·**	**T**	**T**	**A**	**·**	**·**	**·**	**·**	**·**	**T**	**A**	**G**	**·**	**·**	**·**	**T**	**·**	**·**	**·**	**A**	**C**	**T**	**A**	**·**	**·**	**·**	**A**	**T**	**A**	**·**	**T**	**·**	**T**	**·**	**T**	**·**	**G**	**·**	**·**	**·**	**A**	**·**	**·**	**·**	**T**	**·**	**·**	**·**	**·**	**·**	**·**	**·**	**·**	**A**	**·**	**·**	**·**	**A**	**·**	**T**	**·**	**·**	**·**	**·**	**T**	**·**	**A**	**T**	**T**	**A**	**·**	**·**	**T**	**·**	**·**	**·**	**A**	**·**	**·**	**·**	**·**	**·**	**·**	**T**	**C**	**A**	**·**	**·**	**C**	**·**	**T**	**·**	**C**	**C**	**·**	**C**	**C**	**T**	**·**	**C**	**A**	**·**	**·**	**T**	**·**	**C**	**T**	**T**	**A**	**·**	**·**	**·**	**C**	**·**	**·**	**T**	**·**	**·**	**·**	**G**	**·**	**·**	**·**	**·**
*Townsendi*	Colombia	Lyoungi2	**G**	**·**	**·**	**·**	**·**	**G**	**·**	**A**	**·**	**C**	**·**	**A**	**T**	**C**	**A**	**C**	**·**	**·**	**·**	**·**	**·**	**·**	**·**	**T**	**·**	**·**	**·**	**C**	**C**	**G**	**·**	**·**	**·**	**·**	**T**	**T**	**A**	**·**	**·**	**·**	**·**	**·**	**T**	**A**	**G**	**·**	**·**	**·**	**T**	**·**	**·**	**·**	**A**	**C**	**T**	**A**	**·**	**·**	**·**	**A**	**T**	**A**	**·**	**T**	**·**	**T**	**·**	**T**	**·**	**·**	**·**	**·**	**·**	**A**	**·**	**·**	**·**	**T**	**·**	**·**	**·**	**·**	**·**	**·**	**·**	**·**	**A**	**·**	**·**	**·**	**A**	**·**	**T**	**·**	**·**	**·**	**·**	**T**	**·**	**A**	**T**	**T**	**A**	**·**	**·**	**T**	**·**	**·**	**·**	**A**	**·**	**·**	**·**	**·**	**·**	**·**	**T**	**C**	**A**	**·**	**·**	**C**	**·**	**T**	**·**	**C**	**C**	**·**	**C**	**C**	**T**	**·**	**C**	**A**	**·**	**·**	**T**	**·**	**C**	**T**	**T**	**A**	**·**	**·**	**·**	**C**	**·**	**·**	**T**	**·**	**·**	**·**	**G**	**·**	**·**	**·**	**·**
*Townsendi*	Colombia	Lspini	**G**	**·**	**·**	**·**	**·**	**G**	**·**	**A**	**·**	**·**	**·**	**A**	**T**	**C**	**A**	**C**	**·**	**·**	**·**	**·**	**·**	**·**	**·**	**T**	**·**	**·**	**·**	**C**	**C**	**·**	**·**	**G**	**·**	**·**	**T**	**T**	**G**	**A**	**C**	**·**	**·**	**·**	**T**	**A**	**G**	**·**	**·**	**G**	**T**	**·**	**·**	**·**	**A**	**C**	**T**	**G**	**C**	**·**	**·**	**A**	**T**	**A**	**·**	**T**	**·**	**·**	**·**	**T**	**·**	**·**	**·**	**·**	**·**	**A**	**·**	**T**	**·**	**T**	**·**	**·**	**·**	**·**	**·**	**·**	**·**	**G**	**A**	**·**	**·**	**T**	**A**	**·**	**T**	**·**	**·**	**G**	**·**	**T**	**·**	**A**	**T**	**T**	**A**	**·**	**·**	**T**	**·**	**·**	**·**	**A**	**·**	**·**	**·**	**·**	**·**	**·**	**T**	**·**	**A**	**·**	**·**	**C**	**·**	**T**	**·**	**C**	**C**	**·**	**C**	**C**	**T**	**·**	**C**	**A**	**·**	**·**	**T**	**·**	**C**	**T**	**T**	**A**	**·**	**·**	**·**	**C**	**·**	**·**	**T**	**·**	**·**	**·**	**G**	**·**	**G**	**G**	**·**
*Townsendi*	Colombia	Lspini2	**G**	**·**	**·**	**·**	**·**	**·**	**·**	**A**	**·**	**·**	**·**	**A**	**T**	**C**	**A**	**C**	**·**	**·**	**·**	**·**	**·**	**·**	**·**	**T**	**·**	**·**	**·**	**C**	**C**	**·**	**·**	**G**	**·**	**·**	**T**	**T**	**G**	**A**	**C**	**·**	**·**	**·**	**T**	**A**	**G**	**·**	**·**	**G**	**T**	**·**	**·**	**·**	**A**	**C**	**T**	**G**	**C**	**·**	**·**	**A**	**T**	**A**	**·**	**T**	**·**	**·**	**·**	**T**	**·**	**·**	**·**	**·**	**·**	**A**	**·**	**T**	**·**	**T**	**·**	**·**	**·**	**·**	**·**	**·**	**·**	**·**	**A**	**·**	**·**	**T**	**A**	**·**	**T**	**·**	**·**	**G**	**·**	**T**	**·**	**A**	**T**	**T**	**A**	**·**	**·**	**T**	**·**	**·**	**·**	**A**	**·**	**·**	**·**	**·**	**·**	**·**	**T**	**·**	**A**	**·**	**·**	**C**	**·**	**T**	**·**	**C**	**C**	**·**	**C**	**C**	**T**	**·**	**C**	**A**	**·**	**·**	**T**	**·**	**C**	**T**	**T**	**A**	**·**	**·**	**·**	**C**	**·**	**·**	**T**	**·**	**·**	**·**	**G**	**·**	**G**	**G**	**·**
*Townsendi*	Colombia	Ltorvida	**G**	**T**	**·**	**·**	**·**	**·**	**·**	**A**	**·**	**·**	**·**	**A**	**A**	**C**	**A**	**·**	**·**	**·**	**·**	**·**	**·**	**·**	**·**	**T**	**C**	**·**	**·**	**·**	**C**	**·**	**·**	**·**	**·**	**·**	**T**	**T**	**A**	**·**	**·**	**·**	**·**	**·**	**T**	**A**	**G**	**·**	**·**	**G**	**C**	**·**	**·**	**G**	**A**	**·**	**T**	**A**	**C**	**·**	**·**	**A**	**T**	**A**	**·**	**A**	**·**	**·**	**·**	**T**	**·**	**G**	**·**	**·**	**·**	**A**	**·**	**·**	**·**	**T**	**·**	**A**	**·**	**·**	**·**	**·**	**·**	**·**	**·**	**·**	**·**	**T**	**A**	**·**	**T**	**·**	**·**	**·**	**·**	**·**	**·**	**C**	**T**	**T**	**A**	**·**	**·**	**T**	**T**	**·**	**·**	**A**	**·**	**·**	**·**	**·**	**·**	**·**	**T**	**C**	**G**	**·**	**·**	**T**	**·**	**T**	**·**	**T**	**C**	**T**	**C**	**T**	**T**	**·**	**·**	**A**	**·**	**·**	**T**	**G**	**·**	**T**	**T**	**A**	**·**	**·**	**·**	**·**	**·**	**·**	**T**	**·**	**C**	**·**	**A**	**T**	**·**	**·**	**·**
*Townsendi*	Colombia	Ltorvida2	**G**	**T**	**·**	**·**	**·**	**·**	**·**	**A**	**·**	**·**	**·**	**A**	**A**	**C**	**A**	**·**	**·**	**·**	**·**	**·**	**·**	**·**	**·**	**T**	**C**	**·**	**·**	**·**	**C**	**·**	**·**	**·**	**·**	**·**	**T**	**T**	**A**	**·**	**·**	**·**	**·**	**·**	**T**	**A**	**G**	**·**	**·**	**G**	**C**	**·**	**·**	**G**	**A**	**·**	**T**	**A**	**C**	**·**	**·**	**A**	**T**	**A**	**·**	**A**	**·**	**·**	**·**	**T**	**·**	**·**	**·**	**·**	**·**	**A**	**·**	**·**	**·**	**T**	**·**	**A**	**·**	**·**	**·**	**·**	**·**	**·**	**·**	**·**	**·**	**T**	**A**	**·**	**T**	**·**	**·**	**·**	**·**	**·**	**·**	**C**	**T**	**T**	**A**	**·**	**·**	**T**	**T**	**·**	**·**	**A**	**·**	**·**	**·**	**·**	**·**	**·**	**T**	**C**	**G**	**·**	**·**	**T**	**·**	**T**	**·**	**T**	**C**	**T**	**C**	**T**	**T**	**·**	**·**	**A**	**·**	**·**	**T**	**G**	**·**	**T**	**T**	**A**	**·**	**·**	**·**	**·**	**·**	**·**	**T**	**·**	**C**	**·**	**A**	**T**	**·**	**·**	**·**
*Townsendi*	Colombia	Lquasit	**G**	**T**	**·**	**·**	**·**	**·**	**·**	**A**	**·**	**·**	**·**	**A**	**A**	**C**	**A**	**·**	**·**	**C**	**·**	**·**	**·**	**·**	**·**	**T**	**C**	**·**	**·**	**·**	**C**	**·**	**·**	**·**	**·**	**·**	**T**	**T**	**A**	**·**	**·**	**·**	**·**	**·**	**T**	**A**	**G**	**·**	**·**	**G**	**C**	**·**	**·**	**·**	**A**	**·**	**T**	**A**	**·**	**·**	**·**	**A**	**T**	**A**	**·**	**A**	**C**	**·**	**·**	**T**	**·**	**·**	**·**	**C**	**·**	**A**	**·**	**·**	**·**	**T**	**·**	**A**	**·**	**·**	**·**	**·**	**·**	**·**	**·**	**·**	**·**	**T**	**A**	**T**	**T**	**·**	**·**	**·**	**·**	**·**	**·**	**C**	**T**	**T**	**A**	**·**	**·**	**T**	**T**	**G**	**·**	**A**	**·**	**·**	**·**	**·**	**·**	**·**	**T**	**C**	**G**	**·**	**·**	**T**	**·**	**T**	**·**	**T**	**C**	**T**	**C**	**T**	**T**	**·**	**·**	**A**	**·**	**·**	**T**	**G**	**·**	**T**	**T**	**A**	**·**	**·**	**·**	**·**	**·**	**·**	**T**	**·**	**C**	**·**	**A**	**T**	**·**	**·**	**·**
*Townsendi*	Colombia	Lquasit2	**G**	**T**	**·**	**·**	**·**	**·**	**·**	**A**	**·**	**·**	**·**	**A**	**A**	**C**	**G**	**·**	**·**	**·**	**·**	**·**	**·**	**·**	**·**	**T**	**C**	**·**	**·**	**·**	**C**	**·**	**·**	**·**	**·**	**·**	**T**	**T**	**A**	**·**	**·**	**·**	**·**	**·**	**T**	**A**	**G**	**·**	**C**	**G**	**C**	**·**	**·**	**G**	**A**	**·**	**T**	**A**	**C**	**·**	**·**	**A**	**T**	**A**	**·**	**A**	**·**	**·**	**·**	**T**	**·**	**·**	**·**	**·**	**·**	**A**	**·**	**·**	**·**	**T**	**·**	**A**	**·**	**·**	**·**	**·**	**·**	**G**	**·**	**·**	**·**	**T**	**A**	**·**	**T**	**·**	**·**	**·**	**·**	**·**	**·**	**C**	**T**	**T**	**A**	**·**	**·**	**T**	**T**	**G**	**·**	**A**	**·**	**·**	**·**	**·**	**·**	**·**	**T**	**C**	**G**	**·**	**·**	**T**	**·**	**T**	**·**	**T**	**C**	**T**	**C**	**T**	**T**	**·**	**·**	**A**	**·**	**·**	**T**	**G**	**·**	**T**	**T**	**A**	**·**	**·**	**·**	**·**	**·**	**·**	**T**	**·**	**C**	**·**	**A**	**T**	**·**	**·**	**·**
*Serrana*	Peru	Lrobusta	**·**	**·**	**A**	**A**	**T**	**T**	**·**	**C**	**·**	**·**	**·**	**A**	**A**	**·**	**A**	**·**	**·**	**·**	**·**	**·**	**C**	**·**	**A**	**T**	**·**	**C**	**·**	**·**	**·**	**·**	**·**	**·**	**·**	**·**	**·**	**·**	**·**	**·**	**C**	**C**	**T**	**·**	**T**	**A**	**G**	**T**	**·**	**·**	**C**	**·**	**·**	**·**	**A**	**·**	**·**	**A**	**C**	**·**	**·**	**T**	**·**	**A**	**T**	**A**	**T**	**A**	**·**	**T**	**A**	**·**	**·**	**·**	**·**	**·**	**·**	**·**	**C**	**T**	**·**	**·**	**·**	**·**	**A**	**·**	**·**	**G**	**·**	**T**	**A**	**T**	**A**	**·**	**T**	**·**	**C**	**T**	**·**	**·**	**·**	**·**	**T**	**·**	**G**	**T**	**A**	**T**	**T**	**G**	**·**	**A**	**·**	**T**	**·**	**·**	**·**	**·**	**T**	**·**	**A**	**·**	**·**	**T**	**·**	**T**	**·**	**T**	**·**	**·**	**C**	**T**	**T**	**·**	**C**	**A**	**A**	**A**	**T**	**·**	**·**	**T**	**·**	**A**	**·**	**·**	**·**	**·**	**·**	**·**	**·**	**T**	**·**	**·**	**·**	**·**	**·**	**·**	**·**
*Serrana*	Colombia	Lserrana	**·**	**·**	**G**	**A**	**T**	**T**	**·**	**C**	**·**	**C**	**·**	**A**	**·**	**C**	**A**	**·**	**C**	**·**	**·**	**·**	**C**	**·**	**·**	**T**	**·**	**C**	**·**	**·**	**·**	**·**	**·**	**·**	**·**	**·**	**·**	**·**	**·**	**·**	**·**	**C**	**T**	**·**	**T**	**A**	**·**	**·**	**·**	**G**	**C**	**·**	**·**	**·**	**A**	**·**	**T**	**G**	**·**	**·**	**·**	**T**	**·**	**A**	**T**	**A**	**T**	**A**	**·**	**T**	**A**	**G**	**·**	**·**	**·**	**·**	**·**	**·**	**C**	**T**	**·**	**·**	**·**	**·**	**A**	**·**	**·**	**·**	**·**	**T**	**A**	**T**	**A**	**·**	**T**	**·**	**C**	**T**	**·**	**·**	**·**	**·**	**C**	**·**	**G**	**·**	**A**	**T**	**T**	**·**	**·**	**A**	**·**	**T**	**·**	**·**	**·**	**·**	**T**	**·**	**·**	**·**	**·**	**T**	**·**	**T**	**·**	**T**	**·**	**·**	**C**	**T**	**T**	**·**	**·**	**A**	**A**	**A**	**T**	**·**	**C**	**T**	**·**	**A**	**·**	**·**	**C**	**·**	**·**	**T**	**·**	**T**	**·**	**·**	**·**	**·**	**·**	**G**	**·**

**Table 5:** *Cytochrome oxidase* sequence nucleotide composition

Species	Total					Position 1				Position 2				Position 3			
	T	C	A	G	Total	T-1	C-1	A-1	G-1	T-2	C-2	A-2	G-2	T-3	C-3	A-3	G-3
*L. verrucarum*	39.5	17.3	27.2	16.0	667.0	50.2	8.1	39.6	2.1	25.1	17.7	28.4	28.8	43.0	26.1	13.7	17.1
*L. andina*	36.2	19.3	28.5	16.0	657.0	40.9	14.1	43.2	1.8	24.8	17.4	28.4	29.4	42.9	26.5	13.7	16.9
*L. nevesi*	37.0	18.0	28.4	16.6	649.0	44.2	9.7	43.3	2.8	24.1	18.1	28.2	29.6	42.6	26.4	13.4	17.6
*L. serrana*	39.1	16.8	27.9	16.2	667.0	47.1	8.1	41.7	3.1	27.0	16.2	28.4	28.4	43.2	26.1	13.5	17.1
*L. robusta*	38.6	16.7	28.5	16.2	642.0	47.7	7.0	43.5	1.9	25.2	15.9	29.4	29.4	43.0	27.1	12.6	17.3
*L. sauroida*	39.7	16.8	27.1	16.3	667.0	46.6	10.8	39.0	3.6	29.3	13.5	28.4	28.8	43.2	26.1	14.0	16.7
*L. longiflocosa*	39.6	16.8	27.2	16.5	667.0	46.2	10.8	39.5	3.6	29.3	13.5	28.4	28.8	43.2	26.1	13.5	17.1
*L. youngi*	39.1	16.5	28.3	16.2	667.0	46.4	8.1	42.8	2.7	27.5	15.3	28.4	28.8	43.2	26.1	13.5	17.1
*L. spinicrasa*	38.9	16.6	27.5	17.1	667.0	44.8	9.4	40.4	5.4	28.4	14.4	28.4	28.8	43.5	25.9	13.5	17.1
*L. torvida*	39.4	15.9	28.1	16.6	650.5	47.4	7.6	42.1	3.0	27.9	13.7	28.9	29.6	43.0	26.6	13.2	17.3
*L. quasitownsendi*	39.7	16.3	27.7	16.4	667.0	46.9	8.8	41.1	3.4	28.8	14.0	28.4	28.8	43.2	26.1	13.5	17.1
*L. walkeri*	36.6	18.5	29.3	15.8	658.0	41.2	12.5	45.0	1.4	25.6	16.4	29.0	29.0	42.9	26.5	13.7	16.9
Average	38.8	17.0	27.9	16.3	662.0	46.3	9.3	41.4	2.9	27.0	15.5	28.5	29.0	43.1	26.3	13.5	17.1

**Table 6:** Genetic divergence and genetic distance of *L. verrucarum* populations

	Purisima	Huaylas	Lima	Huancavelica	Conchucos	Amazonas	Cajamarca	Piura
Purisima	0.003	0.016	0.015	0.014	0.029	0.023	0.037	0.032
Huaylas	1.4	0.013	0.019	0.019	0.024	0.022	0.029	0.025
Lima	1.4	1.6	0.004	0.003	0.029	0.025	0.039	0.033
Huancavelica	1.6	1.7	0.6	n/a	0.030	0.024	0.040	0.034
Conchucos	2.8	2.2	2.7	2.5	0.005	0.036	0.028	0.029
Amazonas	2.3	2.2	2.4	2.4	2.7	0.003	0.036	0.028
Cajamarca	3.2	2.7	3.4	3.3	2.5	2.8	0.003	0.026
Piura	3.1	2.5	3.2	3.2	2.5	2.6	2.4	0.009

Lower diagonal values represent percent genetic divergence calculated using Kimura 2 parameter. Upper diagonal values represent genetic distance using the model TVM + G + I. The diagonal values represent intrapopulational distance.
